# Health system and household costs of preventive treatment delivery for children exposed to multidrug-resistant tuberculosis in South Africa

**DOI:** 10.1371/journal.pone.0336561

**Published:** 2026-10-08

**Authors:** Thomas Wilkinson, Suereta Fortuin, Anneke C. Hesseling, H. Simon Schaaf, Trinh Duong, Diana M. Gibb, Susan E. Purchase, Lee Fairlie, Suzanne Staples, Neil Martinson, Edina Sinanovic, James A. Seddon

**Affiliations:** 1 Health Economics Unit, School of Public Health, University of Cape Town, Cape Town, South Africa; 2 Division of Molecular Biology and Human Genetics, Stellenbosch University, Cape Town, South Africa; 3 Desmond Tutu TB Centre, Department of Paediatrics and Child Health, Stellenbosch University, Cape Town, South Africa; 4 Innovative Clinical Trials Unit, Institute of Clinical Trials and Methodology, University College London, London, United Kingdom; 5 Wits Research Health Institute, Faculty of Health Sciences, University of the Witwatersrand, Johannesburg, South Africa; 6 THINK, THINK South Africa, Durban, South Africa; 7 Perinatal HIV Research Unit (PHRU) and Infectious Diseases and Oncology Research Institute, University of Witwatersrand, Johannesburg, South Africa; 8 Division of Infectious Diseases, School of Medicine, Johns Hopkins University, Baltimore, United States of America; 9 Department of Infectious Disease, Imperial College London, London, United Kingdom; National Institute of Tuberculosis and Respiratory Diseases, INDIA

## Abstract

**Background:**

Tuberculosis (TB) preventive treatment (TPT) is a core component of TB control. Levofloxacin was recently recommended by the World Health Organization as TPT in children exposed to multidrug-resistant tuberculosis (MDR-TB). However, the affordability and feasibility of TPT provision for governments, health services, individual households and global donors is uncertain.

**Methods:**

This costing analysis used empirical data collected alongside the TB-CHAMP clinical trial which investigated levofloxacin TPT in children exposed to MDR-TB in South Africa. We modelled the societal (health system and household) cost of levofloxacin TPT, other potential future (but not currently recommended) MDR-TB exposure TPT options and compared with drug-susceptible TPT options in children and adolescents under 15 years old. We modelled costs across 12 different medicine and formulation options for 11 paediatric dosing weight-bands using TB-CHAMP and household survey data, with national and international procurement medicine pricing.

**Results:**

Total societal costs of providing levofloxacin TPT and its monitoring per child were US$252 (0- < 5 years) and US$256 (5- < 15-years) and had comparable costs to drug-susceptible TPT regimens when using Global Drug Facility pricing. Pharmaceutical costs for a course of TPT using child-friendly formulations in children <5 years varied between US$38 (levofloxacin), US$80 (bedaquiline) and US$582 (delamanid). Household costs associated with accessing TPT and ongoing monitoring ranged from 1.6%−2.1% of median annual income of affected households, with shorter TPT courses incurring lower household costs. Global Drug Facility procurement achieved TPT pricing at 70% of the costs of national South African procurement, and enabled access to a wider range of child-friendly formulations.

**Discussion:**

Costs of TPT are influenced by factors including medicine pricing and procurement, the use of child-friendly formulations, implementation approach, the duration of treatment, and choice of TPT regimen across weight and age distributions. Costs to households are relatively small but may be significant for impoverished households already managing the financial disruption of a family member with MDR-TB disease.

## Introduction

Each year, an estimated 1.3 million children under 15 years of age develop tuberculosis (TB) worldwide, causing 240,000 deaths, 80% in children under five [1]. Preventing TB in at-risk children is a core pillar of the global TB response [2], and includes identification of child household contacts of people with TB disease, screening for active TB disease, and providing TB preventive treatment (TPT) where appropriate. TPT involves providing a course of antituberculosis antibiotics to lower the risk of developing TB disease; however, achieving universal coverage for children remains a major implementation challenge. Between 2018 and 2022, only 40% of the targeted four million eligible children globally initiated TPT [3].

Multidrug-resistant (MDR)-TB is defined as *Mycobacterium tuberculosis* resistant to at least isoniazid and rifampicin, and while TPT is widely recommended for contacts of patients with drug-susceptible TB, the optimal approach for child contacts of people with MDR-TB has been less certain. Evidence has now emerged from the TB-CHAMP and VQUIN trials. TB-CHAMP, a phase III trial in South Africa, evaluated six months of daily levofloxacin versus placebo in children [4]. V-QUIN, conducted in Viet Nam, assessed levofloxacin for 24 weeks in all age groups [5]. The PHOENIx trial is underway in 12 countries, comparing delamanid with isoniazid TPT in all high-risk household contacts, including children [6]. Additional agents are under investigation including the use of bedaquiline [7,8], and observational studies have demonstrated isoniazid efficacy in child contacts of MDR-TB index patients [9,10]. The findings from TB-CHAMP and V-QUIN have informed the 2024 World Health Organization (WHO) recommendation for a six-month levofloxacin regimen for household contacts of people with MDR-TB [11]. In addition to establishing the most effective anti-TB agents for children, emerging evidence is demonstrating the importance of the type of formulation, palatability, and regimen length as key elements of effective and feasible TB treatment and prevention for children [12,13]. Throughout this manuscript, the term MDR-TB is used to refer broadly to multidrug-resistant or rifampicin-resistant TB (MDR/RR-TB) exposure, as preventive treatment recommendations and programmatic management are generally aligned for contacts exposed to MDR-TB or RR-TB.

Provision of MDR-TB TPT to children has important cost implications. Countries transitioning from donor support face particularly difficult trade-offs, as health investments compete for constrained budgets. Decision makers are also concerned about household costs: although TB services are free from direct cost at the point of care in South Africa and many other settings, accessing treatment can impose a substantial financial burden, especially in households already managing the costs of a family member’s MDR-TB treatment [14]. Although eliminating catastrophic TB-related costs is a main target of the WHO End TB Strategy [15], there is limited evidence on the costs to households associated with accessing MDR-TB prevention and care for children. A household survey called ‘MDRTBkids’ was conducted in South Africa in 2021. Despite free TB treatment at the point of care in South Africa, the study found that the median direct and indirect cost to households for accessing MDRTB treatment for a child was more than US$500 per episode of care, and resulted in more than half of households experiencing catastrophic health expenditure, defined as more than 20% of annual household income [16].

Robust estimates of the costs associated with TPT provision are essential for informing planning, prioritisation, and economic evaluation at national and global level. Although global systematic reviews [17] and standardised cost repositories [18] have improved access to TB cost data, there remains little localised information on the costs of providing MDR-TB TPT, particularly for children. In addition to health system costs, pharmaceutical pricing and formulation availability vary between countries, shaped by national procurement mechanisms. The Global Drug Facility (GDF) offers pooled procurement to secure affordable, quality-assured TB medicines [19]. This is particularly relevant for child-friendly TB medicines, where the market size in any one country may limit the ability to achieve reasonable prices or a range of appropriate formulations. Countries such as South Africa, which procure through national tenders, do not routinely access GDF prices or formulations, which impacts on the expected costs of TPT provision and range of formulations available for children in different age and weight ranges.

While programme implementation costs are important, decision makers also require information on the direct costs incurred by health systems and households when providing TPT to individual children. The objective of this study was to estimate the direct per-patient costs of providing TPT to children aged <15 years exposed to MDR-TB in South Africa from health system and societal perspectives, accounting for differences in weight-based dosing, formulations, and procurement mechanisms.

## Methods

### Study design and analytical approach

This study is an ingredients-based direct costing analysis informed by empirical resource utilisation and household cost data collected alongside the TB-CHAMP trial. The study sources data from the TB-CHAMP trial, the MDRTBkids household survey, normative South African and WHO guidelines, medicine pricing from South Africa’s national procurement programme and the GDF, and South Africa-specific health system costs (Table 1). This study focuses on per-patient costs and is not intended to be a programmatic budget impact analysis. It therefore does not incorporate costs associated with delivering a national-scale TPT programme.

**Table 1:** Parameter cost inputs.

	Value		Reference
MEDICINE COSTS	*Global Drug Facility*	*South African national procurement*	
Levofloxacin 500 mg tablet	US$ 0.05	US$0.13	[26,27]
Levofloxacin 250 mg tablet	US$ 0.03	US$ 0.10	[26,27]
Levofloxacin 100 mg dispersible tablet	US$ 0.12	–	[27]
Isoniazid 100 mg tablet	US$ 0.01	US$ 0.03	[26,27]
Rifampicin 75 mg/Isoniazid 50 mg FDC tablet	US$ 0.05	US$ 0.05	[26,27]
Rifampicin 150 mg/Isoniazid 75 mg FDC tablet	US$ 0.05	US$ 0.06	[26,27]
Rifampicin 150 mg capsule	US$ 0.14	US$ 0.10	[26,27]
Rifapentine 150 mg tablet	US$ 0.30	US$ 0.30	[26,27]
Delamanid 50 mg tablet	US$ 1.48	US$ 1.69	[26,27]
Delamanid 25 mg dispersible tablet	US$ 1.77	–	[27]
Bedaquiline 100 mg tablet	US$ 0.38	US$ 0.61	[26,27]
Bedaquiline 20 mg tablet	US$ 0.43	–	[27]
**HEALTH SERVICES COSTS**			
Standard PHC clinic visit	US$ 12.47		[30,36]
Medicine dispensing fee	US$ 2.60		[29]
Chest X-ray	US$ 66.64		[31]
HIV test	US$ 2.86		[28]
Primary Health Care clinic screening visit: *Clinic visit + Chest X-ray + HIV test + dispensing fee*.	US$ 84.51		Calculated
Primary Health Care clinic TPT visit: *Clinic visit + dispensing fee*	US$ 15.07		Calculated
**HOUSEHOLD COSTS AND INPUTS**			
Average travel cost to visit clinic (round trip)	US$ 0.59		[16]
Average costs incurred at the clinic (food)	US$ 1.01		[16]
Average travel time to visit clinic (round trip)	40 minutes		[16]
Average time spent at clinic per visit	97 minutes		[16]
Minimum wage – South Africa 2025 (per hour)	US$ 1.56		[37]
Time cost of one PHC visit	US$ 3.55		[16]
**EPIDEMIOLOGY AND RESOURCE UTILISATION**			
Proportion that will require HIV testing	97.9%		[4]
Proportion of dispensing clinic visits attended	97.8%		[4]

PHC = Primary Health Care; FDC = Fixed dose combination (tablet).

### Study setting and context

Fig 1 demonstrates the TPT care pathway that informs this costing analysis. It was developed using local guidance [20], informed by the TB-CHAMP protocol, and intended to reflect a pragmatic implementation scenario while excluding trial-specific elements such as additional blood tests.

**Fig 1:**
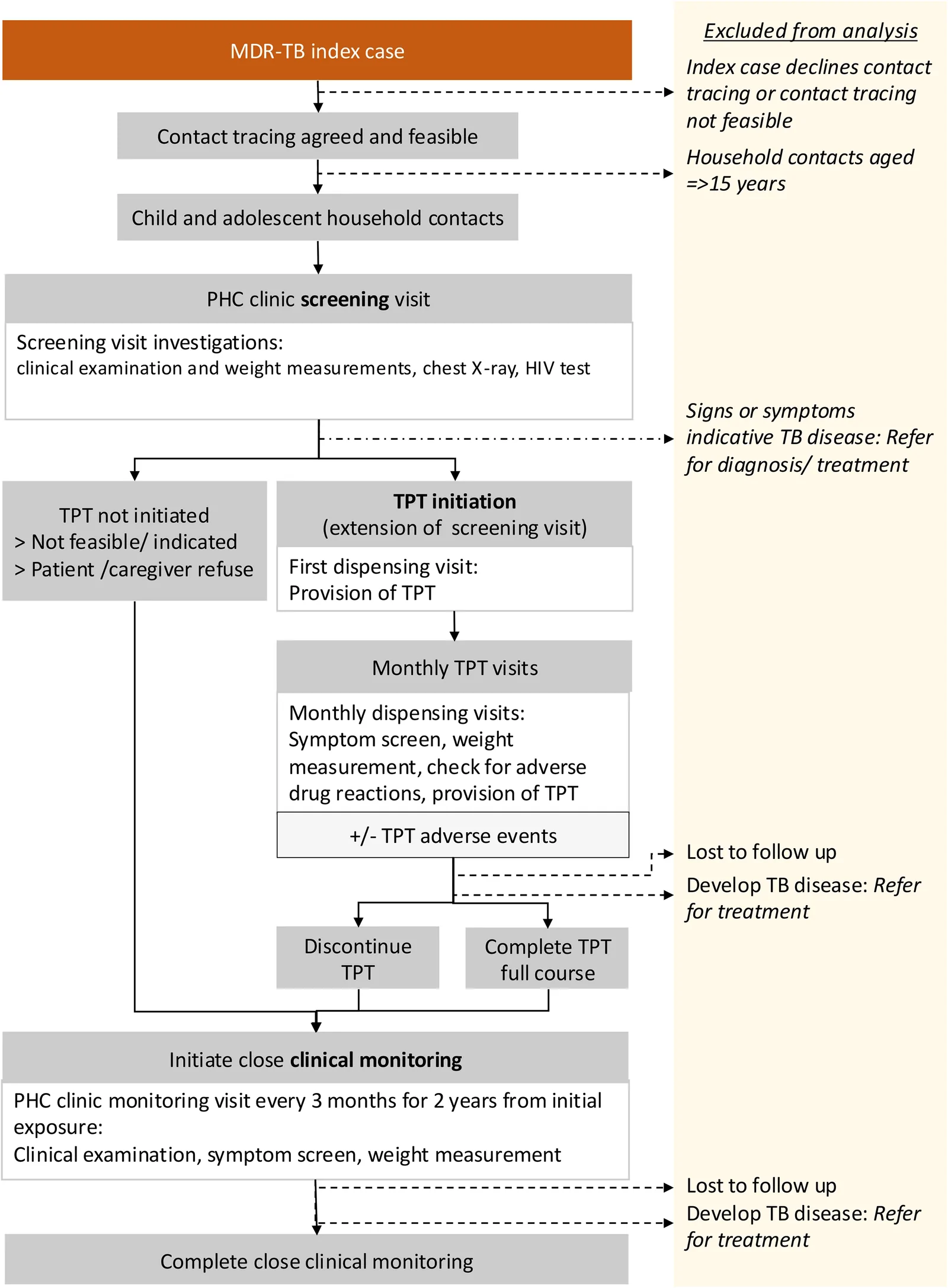
Care pathway of MDR-TB contacts. MDR-TB – Multidrug-resistant tuberculosis; PHC – Primary health care; TPT – Tuberculosis Preventive Therapy.

The care pathway incorporates screening for TB, then either referral for initiation of TB treatment (in case of disease) or offering TPT, and an observation period (Fig 1). An ingredients-based approach was used to incorporate health system and household costs along the care pathway (S1 Appendix, Table A). The TB-CHAMP trial included 89% children below 5 years of age and indicated that levofloxacin was safe in children and demonstrated no statistically significant difference between levofloxacin and placebo at all grades of adverse events [4]. The TB-CHAMP trial also demonstrated that the adult formulation of levofloxacin was well-tolerated by children [21,22]. As TB-CHAMP demonstrated no statistically significant difference in adverse events between levofloxacin and placebo, adverse event-related costs were not incorporated into the base-case analysis. The applicability of this assumption to alternative TPT regimens is uncertain and future analyses should incorporate regimen-specific adverse event costs where empirical data become available.

The analysis represented 12 different regimen and formulation combinations to reflect the range of current and future potential TPT options available in the 0 – < 15-year age group (Table 2). Total per-patient costs for each TPT option were calculated separately for pharmaceutical, non-pharmaceutical health system, and household cost categories.

**Table 2:** Overview of tuberculosis exposure management options.

TB exposure management options	TPT formulation provided	Child-friendly formulation	TPT dosing period (months)	Clinical monitoring period (months)	TPT visits (n)	Clinical monitoring visits (n)
Clinical monitoring only	None	N/A	N/A	24	0	8
***MDR-TB exposure: (current recommendation)***						
6LFX_500 mg tab	Levofloxacin 500 mg tablet	No	6	18	5	4
6LFX_250 mg tab	Levofloxacin 250 mg tablet	No				
6LFX_100 mg disp tab	Levofloxacin 100 mg dispersible tablet	Yes				
6LFX_optimised	Levofloxacin 100 mg dispersible tablet (<25 kg); Levofloxacin 500 mg tablet (25 kg and over);	Partly (100 mg dispersible tab)				
6hH_100 mg tab^1^	Isoniazid 100 mg tablet	Yes	6	18	5	4
***MDR-TB exposure: (Potential future option)***						
6DLM_50 mg tab	Delamanid 50 mg tablet	No	6	18	5	4
6DLM_25 mg disp tab	Delamanid 25 mg dispersible tablet	Yes				
6DLM_optimised	Delamanid 25 mg dispersible tablet (<25 kg); Delamanid 50 mg tablet (25 kg and over);	Partly (25 mg dispersible tab)				
6BDQ_100 mg tab	Bedaquiline 100 mg tablet	No	6	18	5	4
6BDQ_20 mg tab	Bedaquiline 20 mg tablet	Yes				
6BDQ_optimised	Bedaquiline 20 mg tablet (<25 kg); Bedaquiline 100 mg tablet (25 kg and over)	Partly (20 mg dispersible tab)				
***DS-TB exposure:***						
6stdH_100 mg tab^1,^	Isoniazid 100 mg tablet	Yes	6	18	5	4
4R_150 mg cap	Rifampicin 150 mg capsule	No	4	20	3	5
3HR_50/75 mg FDC tablet or 3HR_75/150 mg FDC tablet	Isoniazid 50 mg/ rifampicin 75 mg FDC tablet (<25 kg); Isoniazid 75 mg/ rifampicin 150 mg FDC tablet (25 kg and over)	Yes	3	21	2	5
3HP_100 + 150 mg tab^2^	Isoniazid 100 mg tablet plus rifapentine 150 mg tablet	No	3	21	2	5

BDQ-bedaquiline, DLM-delamanid, FDC-fixed dose combination, hH-high dose isoniazid, stdH-standard dose isoniazid, LFX-levofloxacin, P-rifapentine, R-rifampicin; TPT-tuberculosis preventive therapy. All options receive 1 screening visit. 1. 9H (9 months of isoniazid daily) and 12H (12 months of isoniazid daily) are both recommended TPT options, but 6H (6 months of isoniazid daily) was selected for this analysis as it most closely reflects current clinical practice in South Africa. 2. 3HP is not a recommended TPT option for children weighing <25 kg in the “National Guideline on the treatment of tuberculosis infection in South Africa”, but was included in this analysis as it is a WHO-recommended TPT option for drug-susceptible tuberculosis in children and the South African guideline notes recommendations may change once more safety data on 3HP becomes available.

### Pharmaceutical costing

Numbers of tablets or capsules were calculated according to the weight-based harmonised dosing recommended in the WHO consolidated guidelines on TPT for standard-dose isoniazid, rifapentine, rifampicin, levofloxacin [23] and the South African guidelines for delamanid and high-dose isoniazid [20]. Recommended therapeutic treatment doses were used to reflect TPT dosing for bedaquiline [24]. We assumed medicines were dispensed for 28 days at a time, and that the required dose administered was rounded up to the nearest whole tablet, or half tablet where tablets are scored or are dispersible [25]. As older children may be more able to tolerate a solid dosage tablet formulation, we modelled an “optimised” regimen using the dispersible tablet in the lower weight bands (<25 kg), and solid dosage in the higher weight bands. The rate of compliance with scheduled medicine dispensing and monitoring visits observed in the TB-CHAMP trial was used to estimate expected medicine utilisation and associated costs. The reported medication adherence (defined as proportion of prescribed tablets consumed) in the TB-CHAMP trial was high (96%; interquartile range 90%−99%) with 73% of participants taking more than 90% of prescribed doses, and no significant difference between adherence for levofloxacin compared to placebo [4]. The rate of attendance to medicine-dispensing visits in TB-CHAMP (97.8%) was applied to the calculated cost of all TPT regimens in this analysis to reflect the expected under-use of medicine in an implementation scenario, recognizing that once a monthly prescription is dispensed, the total of the cost of the medicine is incurred (under an assumption that a medicine that is dispensed but not consumed is wasted), but non-dispensed medicine does not incur a cost.

Unit costs of medicines were calculated using prices listed in South Africa’s public sector national procurement system October 2025 [26] and the GDF September 2025 [27], costs of laboratory tests were from the National Health Laboratory Service (NHLS) state pricing list (current version published in 2023 and are current in year 2025) [28], dispensing costs were sourced from the Uniform Patient Fee Schedule (UPFS) (April 2025) [29] and clinic visit and chest x-ray unit costs were derived from existing literature [30,31]. Expected frequency of routine clinic visits was calculated based on South Africa’s national TB guidance [20].

### Non-pharmaceutical health system costing

Non-medicine health system costs were determined by the type and number of contacts with the health service for each TPT option. All options included a screening visit, which consisted of a standard clinic visit (for symptom screening, weight measurement, physical examination), plus a chest x-ray, HIV test (if status unknown), and dispensing of the first month of TPT (except for the non-TPT option). Under TB-CHAMP, a test of TB infection was not required in children < 5 years of age and in older children (with and without HIV) a test of TB infection was required. Current TB programme resource constraints mean that tests of TB infection are not routinely provided in South Africa (and therefore costs are not incurred) in those 5 years of age and older who are exposed to TB. Therefore, the base case costs represented in this analysis do not incorporate TST testing for any age groups. The cost of introducing TST for children 5 years old and above is provided as an illustrative scenario and should be considered in cost effectiveness analysis using effect sizes as demonstrated in the TB-CHAMP trial. Monthly TPT dispensing visits consisted of a standard clinic visit, checking for adverse drug reactions, weight gain and dispensing of TPT medicines and adjusting doses as required. Clinical monitoring visits consisted of a standard clinic visit and were estimated to take place every three months up to two years from initial exposure once the TPT course was completed, or monthly for six months followed by every three months for two years, if no TPT was provided. Monitoring costs were included as a defined costing scenario informed by the TB-CHAMP protocol and local guidance; actual monitoring practices may vary between TB programmes. Healthcare costs associated with TPT provision excluded procedures and interventions required for the identification and management of TB disease and/or those required for the management and monitoring of HIV.

### Household costing

The household costs analysis utilises data collected from the MDRTBkids survey, a concurrent costing survey that included data from 45 households with a child with MDR-TB disease in the Western Cape, South Africa, who routinely initiated treatment between 1 January 2018–31 August 2021 [16]. The costs incurred by households to access TPT and monitoring included travel costs, expenses while visiting facilities and time costs of attending clinics, and these were uplifted to 2025 costs by applying the annual South African consumer price index [32]. The analysis estimated household costs by applying average per-instance cost of attending clinic visits and collecting prescribed medicines as observed in the MDRTBkids survey, multiplied by the expected health system interaction of the different TPT management strategies. The MDRTBkids survey population was predominantly urban/peri-urban and largely reflected costs associated with accessing primary healthcare facilities in the Western Cape, South Africa. Household costs may therefore differ in more rural settings, where transport distances, travel time, and access barriers may be greater. While the number of dispensing and clinic visits varied between some strategies, it was assumed that per-clinic visit costs did not differ between TPT regimens – the same monthly collection cost would be incurred by households regardless of whether it was to collect a prescription for levofloxacin or for delamanid. The total average household costs of accessing TPT over the treatment and monitoring duration was represented as a proportion of average household income of participants in the MDRTBkids survey population, under an assumption that average monthly income of a household with a child with MDR-TB disease would be comparable to a household with a child who is exposed to MDR-TB.

### Currency and inflation adjustments

Costs collected from published literature were converted to South African Rand (ZAR) in the year of analysis. When relevant, costs were inflated to 2025 using the South African consumer price index [32]. Results were developed in ZAR and US$ and are presented in the main text for an international audience in US$ without adjustment for purchasing power parity.

### Ethics

The TB-CHAMP trial – the protocol of which included this analysis, was approved by the Stellenbosch University Health Research Ethics Committee (M16/02/009) and the University of the Witwatersrand Ethics Committee (160409) and the South African Health Products Regulatory Authority (SAHPRA). The MDRTBkids study received ethics approval from the Stellenbosch University Health Research Ethics committee (N20/09/102).

## Results

The pharmaceutical costs per-patient by TPT regimen, formulation, age range, and procurement mechanism are shown in Table 3, the corresponding costs per weight band are shown in S1 Appendix Table C. Fig 2 shows levofloxacin 6-month TPT regimen cost by weight, with weighted average across the 0- < 15-years age range.

**Table 3:** Pharmaceutical cost of TPT per patient by regimen, procurement mechanism and age cohort.

	GDF price ($)				National price ($)			
TPT regimen	Age 0- < 2	Age 2- < 5	Age 0- < 5	Age 5- < 15	Age 0- < 2	Age 2- < 5	Age 0- < 5	Age 5- < 15
6LFX_500 mg tab	$3.72	$5.31	$4.67	$7.84	$11.00	$15.70	$13.82	$23.18
6LFX_250 mg tab	$4.36	$5.35	$4.95	$8.65	$15.81	$19.41	$17.97	$31.41
6LFX_100 mg disp tab	$31.98	$42.45	$38.26	$79.03	–	–	–	–
6LFX_optimised	$31.98	$42.33	$38.19	$21.27	$31.98	$42.36	$38.20	$32.47
6DLM_50 mg tab	$243.22	$243.22	$243.22	$552.65	$278.24	$278.24	$278.24	$632.21
6DLM_25 mg disp tab	$581.79	$581.79	$581.79	$1,321.93	–	–	–	–
6DLM_optimised	$581.79	$581.12	$581.39	$648.86	$581.79	$581.19	$581.43	$718.48
6BDQ_100 mg tab	$29.67	$31.89	$31.01	$45.59	$47.99	$51.58	$50.14	$73.73
6BDQ_20 mg tab	$57.64	$94.69	$79.85	$233.45	–	–	–	–
6BDQ_optimised	$57.64	$94.37	$79.66	$69.02	$57.64	$94.42	$79.69	$91.04
6hH_100 mg tab	$2.84	$4.10	$3.59	$7.17	$6.96	$10.05	$8.81	$17.57
6stdH_100 mg tab	$2.01	$2.89	$2.54	$4.31	$4.93	$7.08	$6.22	$10.57
4R_150 mg cap	$15.55	$16.08	$15.87	$44.93	$10.92	$11.29	$11.14	$31.56
3HR_FDC tab	$10.64	$15.28	$13.42	$15.07	$10.53	$15.12	$13.28	$16.53
3HP_100 mg + 150 mg tab	$6.13	$8.92	$7.80	$17.80	$6.52	$9.48	$8.30	$18.99

BDQ-bedaquiline, DLM-delamanid, H-isoniazid, hH-high dose isoniazid, stdH-standard dose isoniazid, LFX-levofloxacin, P-rifapentine, R-rifampicin.

*All TPT regimen formulations available under Global Drug Facility (GDF) procurement. Where a formulation is not listed under National Procurement, it is not available in South Africa under the national procurement mechanism (the Master Health Product List (MHPL)).

**Fig 2:**
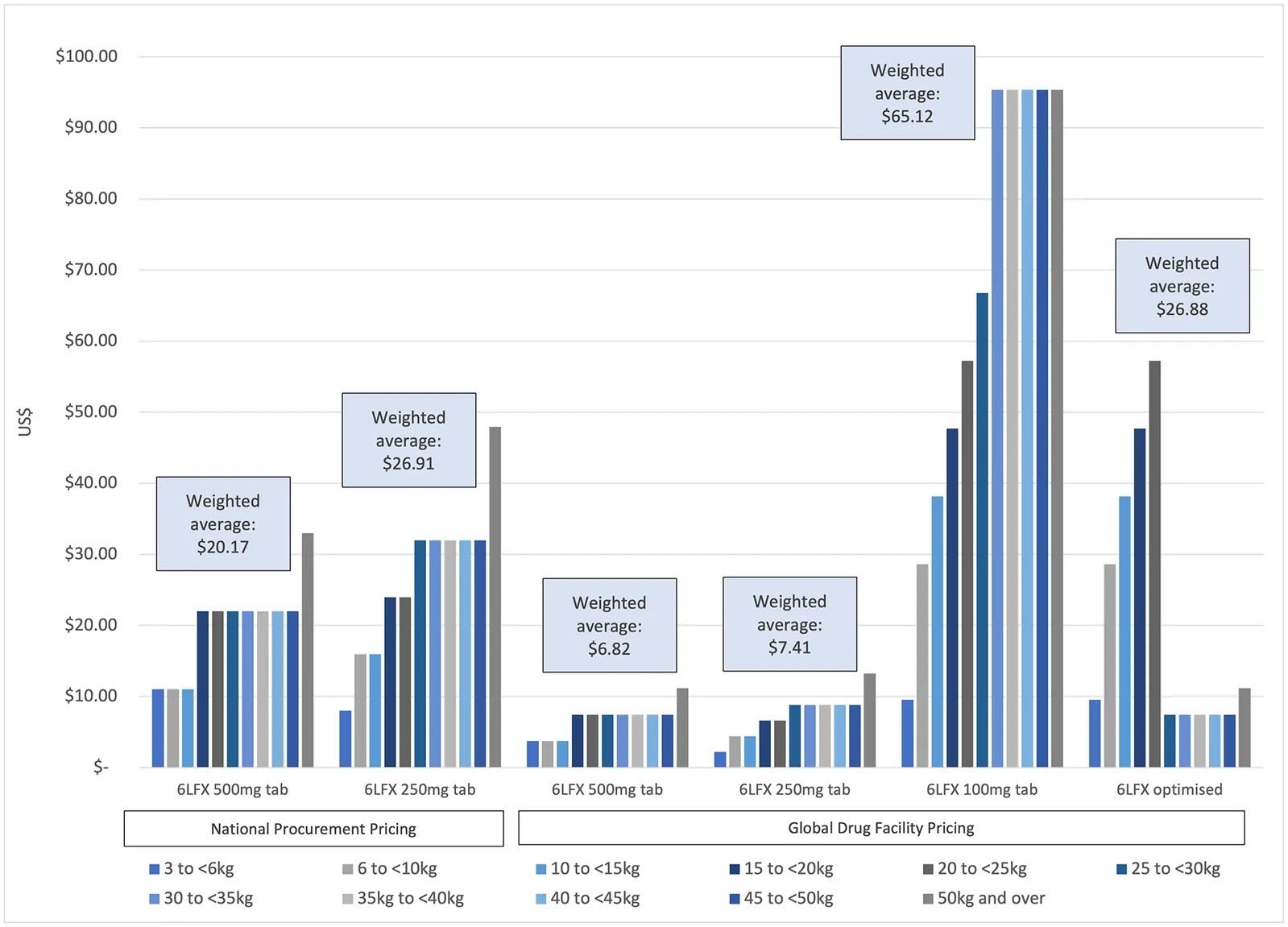
Medicine cost by weight band for 6-month levofloxacin-containing TPT options, with weighted average across the 0- < 15-year-old age range. LFX-levofloxacin. The levofloxacin 100 mg dispersible tablet formulation not currently available under South Africa national procurement mechanism. Under optimised regimen: children weighing <25 kg children receive dispersible formulation, children weighing 25 kg and over receive solid dosage formulation.

There is substantial variation in cost between TPT regimens and between age and weight cohorts. Levofloxacin TPT regimen containing 250 mg and 500 mg solid non-dispersible tablets have a comparable cost to drug-susceptible TPT regimens across age ranges. Under GDF pricing, a TPT course using the 100 mg dispersible tablet formulation of levofloxacin is approximately 8-fold the price of the 250 mg tablet ($5 vs $38 in the 0- < 5-year age range, and $79 vs $9 in the 5- < 15-year age range). The levofloxacin dispersible tablet formulation is currently not yet purchased under South African national procurement, and the principal formulation of interest as used in the TB-CHAMP trial is the levofloxacin 250 mg solid dosage tablet. However, the optimised regimen reflected in Table 3 modelled the cost if the South African national programme obtained the levofloxacin 100 mg dispersible tablet at GDF pricing. This demonstrated that if South Africa was able to access GDF supply of dispersible levofloxacin formulations only, and restricted use to lower age ranges, there would only be a marginal increase in costs compared to all children using the solid dosage levofloxacin formulation.

Potential future TPT regimens of delamanid and bedaquiline, if proven to be efficacious and safe, currently have substantially higher cost than levofloxacin, particularly under South African national pricing and for dispersible tablet formulations. However, future generic market entry, changes in procurement mechanisms, and evolving global pricing strategies may substantially reduce unit costs over time. Future costing analyses and policy recommendations should therefore incorporate up-to-date pricing information.

Total societal costs of TPT provision, incorporating pharmaceutical costs, other health system costs, and household costs for the 0- < 5-year age range are shown in Fig 3 (with tabulated data and other age ranges in S1 Appendix Tables F, G, H, I). Across all TPT regimens, total societal costs ranged from US$210 to US$829 per child completing treatment and follow-up. Levofloxacin-containing regimens were associated with total societal costs of approximately US$252–285 per child depending on formulation and procurement mechanism, while the highest total costs were observed for delamanid-containing regimens due to substantially higher pharmaceutical prices.

**Fig 3:**
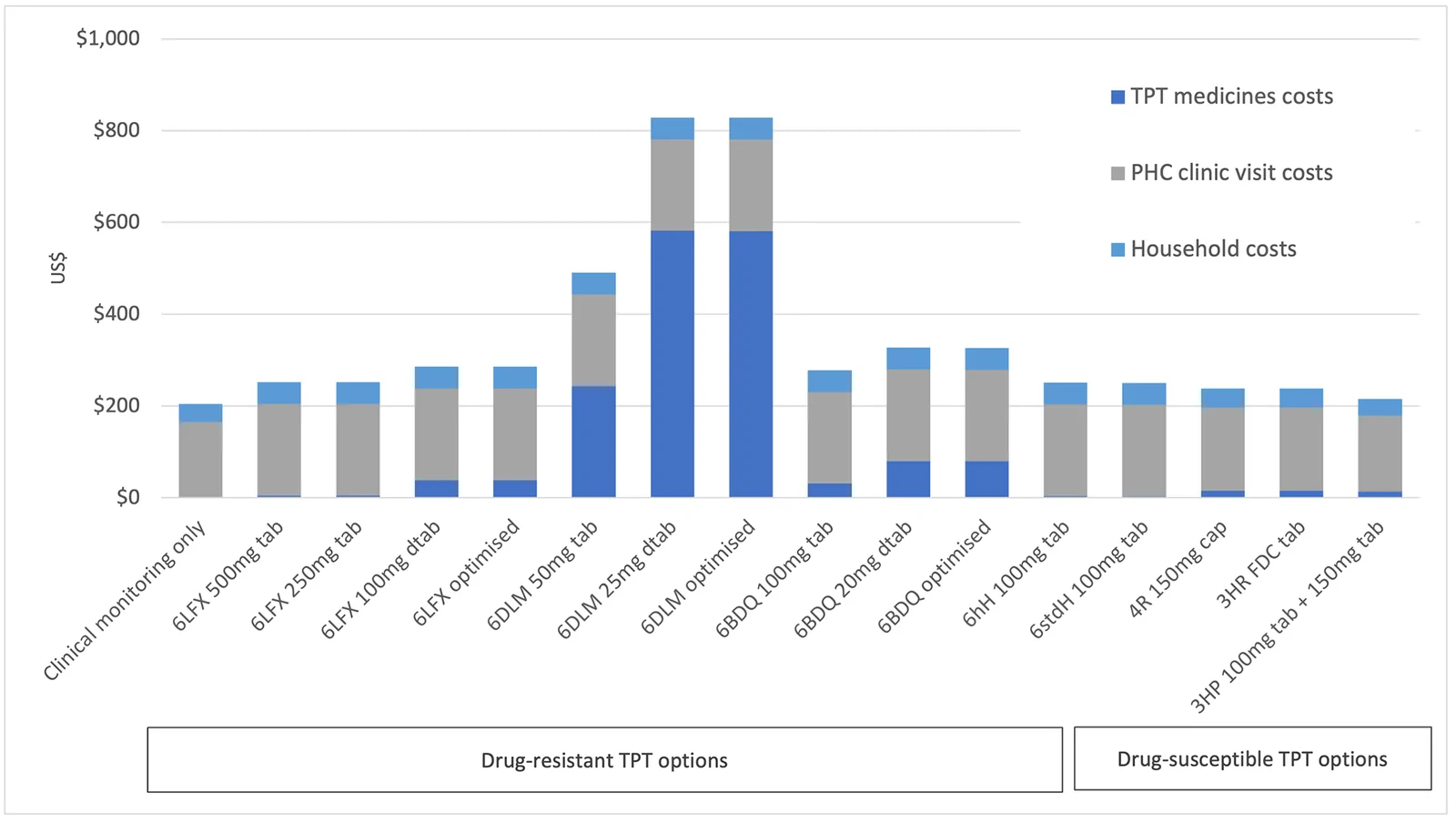
Total mean societal costs (US$) by TPT regimen and formulation for children 0- < 5 years of age. BDQ = bedaquiline; DLM = delamanid; hH = high dose isoniazid; stdH = standard dose isoniazid, LFX = levofloxacin, P = rifapentine, R = rifampicin; tab = solid dosage tablet formulation; dtab = dispersible tablet formulation; FDC = fixed-dose combination tablet; TPT = tuberculosis preventive therapy. Under optimised regimen: children weighing <25 kg children receive dispersible formulation, children weighing 25 kg and over receive solid dosage formulation.

As an illustrative scenario, if 10% of children experienced an adverse event requiring one additional primary healthcare clinic visit, the expected health system cost of levofloxacin TPT would increase by approximately US$1.25 per child (0.6% of direct health system costs of delivering TPT). This suggests that minor adverse events requiring limited additional healthcare utilisation are unlikely to materially affect overall cost estimates. More severe adverse events requiring hospitalisation would have a greater impact on costs; however, estimation of these costs would require robust evidence regarding both the frequency and severity of adverse events when these medicines are used as TPT regimens.

Pharmaceutical costs represent the largest variation between options (ranging from $5 to $582 in the 0- < 5-year age range). Non-pharmaceutical health system costs were relatively stable between options, ranging from US$165 for clinical monitoring alone to US$200 for a 6-month TPT regimen with monitoring. (S1 Appendix Table I). Non-pharmaceutical health system costs are predominantly driven by clinic visits, differentiated by visits associated with dispensing or monitoring, with regimen length determining overall costs. The analysis does not incorporate costs of commercial Interferon-Gamma Release Assays or tuberculin skin testing as part of the pathway, as these are not required for initiation of TPT in current South African guidance [25]; and were not required in the TB-CHAMP protocol for the < 5 age group. As an illustrative scenario, inclusion of an IGRA or TST in the screening pathway would add approximately US$110 or US$38 per child [28], respectively, to health system costs. As these costs are additive and independent of regimen selection, the proportional impact would be similar across all TPT options.

Monitoring costs comprised four scheduled primary care clinic visits per child and were assumed to be identical across all tuberculosis preventive treatment regimens. As implementation of TPT under routine programmatic conditions may involve less intensive follow-up than assumed in this analysis, reducing monitoring from four to two primary care clinic visits would be expected to reduce total health system costs by approximately 12%, demonstrating that pharmaceutical and regimen-related costs remain the principal drivers of overall cost variation.

Household costs ranged from US$37-US$48 across the whole treatment and monitoring period and were a small proportion of overall costs. Household costs were largely driven by duration of TPT regimen and were broadly consistent across regimens. Households participating in the TB-CHAMP trial reported a median total monthly income of US$186 in 2025 values (South African Rand (ZAR) 3,420), indicating that TPT costs represented 1.6%−2.1% of annual income in affected households over the treatment and monitoring period.

## Discussion

This analysis details the cost of provision of a TPT strategy for children <15 years old exposed to MDR-TB in the South African context from a health system and household perspective. We demonstrate that factors including regimen choice, medicine formulation type, weight of the child, and procurement mechanism have a significant impact on the per-patient pharmaceutical costs. The analysis combines trial data for key parameters including pharmaceutical usage, clinic visits and weight distribution of participants, with normative recommendations on treatment regimens for medicines not included in the TB-CHAMP trial.

This analysis provides further context to existing work. A global modelling study by Dodd and colleagues assessed the cost effectiveness of TPT in household contact management in children and included regimen options that are represented in this analysis [33]. Dodd and colleagues used cross-country costs estimates based on GDF pricing for pharmaceuticals and per-country costs from WHO and generalised estimates based on GDP modifiers. An analysis by Jo and colleagues [34] assessed the costs and cost-effectiveness of scale up of short-course TPT in child contacts of people with drug-susceptible TB across 12 countries, which also used global estimates modified across jurisdictions and secondary data from the Global Health Cost Consortium. Our analysis broadly aligns with the estimates from these studies, but provides further detail regarding the impact of different formulations, regimens, and weight bands on costs. Unlike many economic evaluation models, the majority of cost inputs used in this analysis were derived from observed prices and tariff schedules rather than estimated parameters. Consequently, variation in results was driven primarily by structural factors, including procurement mechanism, formulation choice, regimen selection, and child weight distribution. We provide contemporaneous localised health system costs, and use trial-based utilisation and adherence data rather than relying solely on normative assumptions. This analysis also provides current pharmaceutical and other costing inputs for the year 2025; there has been substantial reduction in the costs of key antituberculosis agents (particularly bedaquiline and delamanid) in the previous three-year period.

Non-pharmaceutical health system costs were predominantly attributable to clinic visits for monitoring and dispensing and constituted a substantial proportion of the costs, but were largely unaffected by regimen choice. Clearly shorter regimens and less intensive monitoring would reduce costs. Additional investigations at screening would substantially increase costs. A single IGRA test may provide useful additional information but would increase the health system costs per patient by 60%, far exceeding the cost of pharmaceutical treatment. This additional cost, together with practical constraints on providing IGRA testing at scale, supports the WHO guidance to initiate TPT for at-risk children below 5 years of age without the requirement for testing by TST or IGRA [25].

The household costs of providing TPT constitute the time to access clinics for monitoring and prescription pickup. These are relatively small, particularly relative to expected household costs of accessing treatment for tuberculosis disease. However, these costs may be a significant burden to households that may already be financially vulnerable. Importantly, household costs are not met by the national TB programme budget, but by general household expenditure. The MDRTBkids household survey established that for many households, a family member is able to walk to the local primary care clinic, avoiding direct travel costs. However, travel time and waiting time at clinics still represent a substantial opportunity cost for households.

Weight variation has a significant impact on per-patient costs due to weight-based dosing, with differences in costs substantially higher for patients in the < 5 kg weight bracket (generally less than 2 months old) compared to children in the 20–25 kg weight bracket (approximately 4 or 5 years old), with some variation by type of formulation. The inclusion of “optimised” regimens in the analysis attempted to reflect a pragmatic implementation scenario. Children in the lower weight ranges (<25 kg) were assumed to receive dispersible (lower mg dose of medicine per unit) formulations, and those above were assumed to receive adult formulations, reducing the average cost of TPT across the age cohort. While this analysis reflects TB-CHAMP weight-based dosing for levofloxacin, the recent harmonization of weight banding across tuberculosis treatment and prevention by WHO will enable further consolidation of dose schedules [23].

The analysis demonstrates that for levofloxacin, the use of a child-friendly formulation (100 mg dispersible tablet) is likely to be more expensive than the adult 250 mg or 500 mg solid-dosage formulations. While this proportional price difference may be significant for national TB programmes at scale, dispersible formulations may offer implementation advantages not captured in this costing analysis, including improved palatability, easier administration for caregivers, and more accurate weight-based dosing without the need to crush or split tablets [21,22]. These factors may also influence acceptability and treatment use in routine programmatic settings. If South Africa were to procure (or receive through donor support) the 100 mg levofloxacin formulation, the unit price would be an important consideration in the sustainability and scalability of a national TPT programme for children exposed to MDR-TB.

The differences in prices secured through the national South African tender and the GDF are an important driver of pharmaceutical costs. The GDF procures the levofloxacin 250 mg tablet at 28% of the price of the South African national procurement process, and across all formulations assessed in this analysis, GDF pricing was 70% of that achieved through South African procurement prices. It is expected that over time, the price of both delamanid and bedaquiline will reduce further following entry of generic manufacturers and ongoing competitive processes. Any policy decisions by South Africa’s national TB programme and Essential Drugs Programme related to use of levofloxacin or other regimens for TPT or MDR-TB disease treatment should take pricing and procurement dynamics into account, and particular consideration should be given to South Africa accessing GDF supply, particularly the 100 mg dispersible tablet. Realisation of the lower prices observed through the GDF would depend on national procurement policies and decisions regarding the use of international pooled procurement mechanisms. While South Africa currently relies primarily on national competitive tender processes, future procurement reforms, strategic purchasing arrangements, or partnerships with international procurement mechanisms could alter the availability of these prices to the national TB programme.

This analysis is purposefully South African based, drawing on empirical data from the TB-CHAMP trial, the MDRTBkids local household survey, and localised South African prices. The analysis models GDF pricing in addition to South African national tender pricing to facilitate transferability of the results, in particular the pharmaceutical costs to other national TB programmes that access GDF pricing. The transfer of total costs, however, would require modification of localised costs of screening, dispensing and primary health clinic monitoring visits.

The analysis has some limitations. Most costs are based on trial or current real prices (such as the South African tender and national laboratory price list); however, costs based on non-empirical data such as costs of primary care clinic visits required secondary data and inflation of reported costs to reflect 2025 prices. As with all costing analysis, prices, and particularly pharmaceutical prices, are subject to change associated with procurement cycles, and should be interpreted with reference to dates of cited pricing sources.

In line with established costing practice, costs specific to the operational aspects of the TB-CHAMP trial were excluded, as these would not be replicated under routine programme implementation. Although trial-specific operational costs were excluded, participation in a clinical trial may influence adherence, visit attendance, and treatment completion through mechanisms that are difficult to quantify, including enhanced participant support, follow-up, and engagement with dedicated research staff. These factors should be considered when applying the cost parameters reported here to routine programmatic settings.

Our analysis reflects current MDR-TB TPT clinical guidance with potential future regimens for comparison. Costs may significantly decrease with the introduction of shorter regimens, options with fewer follow-up or monitoring visits, and further competition in the market for key proprietary pharmaceuticals. Household costs may also differ substantially between urban and rural settings. The MDRTBkids survey informing household cost estimates was predominantly urban/peri-urban, and costs associated with accessing TPT in rural communities may be higher due to increased transport requirements, travel time, and reduced healthcare accessibility. Household costs were estimated using per-visit costs derived from the MDRTBkids survey. The time and opportunity costs associated with TPT visits may differ from those associated with MDR-TB treatment visits, although any resulting changes in estimated household costs would be expected to vary proportionally with the assumed per-visit cost.

Although informed by empirical utilisation data, the analysis does not fully reflect all aspects of routine programmatic implementation. The empirical data are from a clinical trial, so may overestimate adherence to treatment over the 6-month period or feasibility of the health system to provide monitoring over the 2-year period. However, the data may be more reflective of actual implementation compared to a completely normative ingredients-based costing analysis that implicitly assumes 100% compliance with recommended guidance. This analysis also did not represent cost efficiencies that may be achievable with integration of TPT with other programmes to dispense medication or monitor patients (such as antiretroviral treatment). It is expected that it would be possible for household costs to be reduced if a family member was able to collect medication for both the member receiving treatment for MDR-TB and the child’s TPT at the same visit.

Our analysis excludes some cost elements that may be relevant to some users. The “above service delivery” costs, such as the managerial costs of organising the TPT programme have not been included but may reasonably be considered to fall within existing functions of the TB programme and health managers. Importantly, the “above service delivery” costs are not expected to vary significantly between different TPT options. Contact tracing and case-finding activities required to identify children eligible for TPT were not included in this analysis. These activities can incur substantial human resource and programme costs, including community health workers, administrators, and clinicians, and should be incorporated when assessing the costs of a wider TB prevention programme.

Medicine administration of adult formulations (such as a solid tablet) for children in this age group commonly involves crushing the tablet and suspending in water, yoghurt, or other palatable media; administration of dispersible formulation involves suspending the tablet in water. The health professional or caregiver costs associated with administration of the different formulations were not included in the analysis due to uncertainty associated with estimation and attribution of costs; however, it is likely that the convenience of the dispersible formulations represent slightly lower administration cost. Future household cost and acceptability studies could usefully explore the time, effort, and resource implications associated with administering different paediatric formulations, as these factors may influence both caregiver burden and treatment acceptability.

The analysis did not include downstream costs associated with the diagnosis and treatment of TB disease. Inclusion of these costs would require modelling the full clinical pathway and associated health outcomes following MDR-TB exposure and is therefore more appropriately addressed within a cost-effectiveness analysis than a direct costing study.

As the TB-CHAMP trial, which included almost 1000 children, did not demonstrate a significant difference in adverse events between levofloxacin and placebo, the cost estimates in this analysis do not incorporate costs to health systems or households of adverse event management. In addition, the adverse event experience observed in TB-CHAMP was derived primarily from children aged <5 years and may not be generalisable to older children and adolescents. Subsequent pooled analysis of the TB-CHAMP and V-QUIN trials found that serious and grade 3 or higher adverse events were uncommon and did not differ significantly between levofloxacin and placebo overall, although adverse events resulting in treatment discontinuation and musculoskeletal adverse events were more frequently reported in older age groups, particularly adolescents and adults. Future analyses incorporating adverse event costs should therefore consider age-specific and regimen-specific evidence where available [35].

In conclusion, this is the first analysis that utilises empirical data and localised costs and prices to estimate the expected per-patient costs of TPT for MDR-TB in South African children, across a range of potential treatment options. The results will contribute to existing initiatives to collate and disseminate TB cost parameters [17]. The findings will be important inputs to future cost-effectiveness analyses, budget impact assessments, and planning of TPT services.

## Supporting information

S1 Appendix Supplementary Tables and Figures. Tables and Figures supporting main manuscript.
(DOCX)
