# Supplementary material for: Health system and household costs of preventive treatment delivery for children exposed to multidrug-resistant tuberculosis in South Africa
Source: PLoS One. 2026 Oct 8;21(10):e0336561. doi: 10.1371/journal.pone.0336561 (PMC13648985; doi:10.1371/journal.pone.0336561)
Supplement: Supplementary file 1 [file pone.0336561.s001.docx]

Health system and household costs of preventive treatment delivery for child exposed to multidrug-resistant tuberculosis in South Africa

S1 Supplementary Appendix

[Table A: South African national guidelines with recommendations on the management of child household contacts of patients with drug-resistant TB with TPT	2](#_Toc240469877)

[Table B: Additional parameter inputs:	4](#_Toc240469878)

[Table C: Summary of patient pathway for TPT provision to household contacts:	4](#_Toc240469879)

[Table D & Table E Weight banding in TB-CHAMP	5](#_Toc240469880)

[Table F: Health system costs (US$) by TPT regimen for children aged 0-<15 years (excluding pharmaceutical costs)	6](#_Toc240469881)

[Table G: Household costs (US$) (non-health system costs) by TPT regimen for children aged 0-<15 years	6](#_Toc240469882)

[Table H: Pharmaceutical cost per patient (US$) completing TPT by regimen, formulation, weight band, and procurement mechanism	7](#_Toc240469883)

[Table I: Total societal costs of different TPT regimen and formulation ($US)	8](#_Toc240469884)

[Figure A: Average pharmaceutical costs across weight bands for children <5 years for TPT regimen and formulation by procurement mechanism	9](#_Toc240469885)

[Figure B: Pharmaceutical cost (US$) of TPT regimen and formulation by weight for children <5 years of age using Global Drug Facility procurement pricing	10](#_Toc240469886)

# Table A: South African national guidelines with recommendations on the management of child household contacts of patients with drug-resistant TB with TPT

| Current Guidance | | |
| --- | --- | --- |
| **National guideline** | **Year of publication** | **Recommendation** |
| Standard Treatment Guidelines and Essential Medicines List for South Africa: Hospital level paediatrics (2023 edition) (1) | 2023 | Preventive therapy in case of drug-resistant TB contact:  Isoniazid mono-resistance (rifampicin susceptible):   - Rifampicin, oral, 15 mg/kg daily for 4 months.   Rifampicin mono-resistance (isoniazid susceptible):   - Isoniazid, oral, 10 mg/kg daily for 6 months.   MDR-TB with second line sensitivity:   - Levofloxacin 15-20mg/kg daily for 6 months (maximum dose: 1000mg) - High-dose isoniazid, oral, 15-20mg/kg daily for 6 months (maximum dose: 600mg)   Refer case if simplification of prophylaxis regimen is required  XDR-TB:   - Close follow-up for two years. - Ensure household infection control practices are observed.   Refer all cases. |
| National guidelines on the treatment of tuberculosis infection (2) | 2023 | For the management of DR-TB exposure, refer to “the latest National DR-TB Guidelines” |
| Superseded guidance: | | |
| **National guideline** | **Year of publication** | **Recommendation** |
| Management of rifampicin-resistant tuberculosis: A clinical reference guide (3) | 2019 | Rifampicin mono-resistant TB:   - 6 months isoniazid   Isoniazid mono-resistant:   - 4 months rifampicin   Rifampicin-resistant TB:   - 6 months of a fluoroquinolone either alone or in combination with one or two other agents (ethambutol, high-dose isoniazid)   MDR-TB with FLQ resistance:   - High dose isoniazid or consider delamanid with input from National Clinical Advisory Committee |
| Standard Treatment Guidelines and Essential Medicines List for South Africa: Hospital level paediatrics (2017 edition) (4) | 2017 | Preventive therapy in case of drug-resistant TB contact:  Isoniazid mono-resistance:   - Rifampicin, oral, 15 mg/kg daily for 4 months.   Rifampicin mono-resistance:   - Isoniazid, oral, 10 mg/kg daily for 6 months.   MDR-TB:   - High-dose isoniazid, oral, 15-20mg/kg daily for 6 months 300mg - Ethambutol, oral, 20-25mg/kg daily for 6 months 400mg - Levofloxacin 15-20mg/kg daily for 6 months 500mg   Refer case if simplification of prophylaxis regimen is required  XDR-TB:   - Close follow-up for two years. - Ensure household infection control practices are observed. - Refer all cases. |
| National tuberculosis management guidelines 2014 (5) | 2014 | - All asymptomatic close contacts of MDR-TB patients should be screened at six monthly intervals for up to two years. - HIV positive contacts should be followed-up 3 monthly and if active MDR-TB develops, referred immediately for treatment. - Asymptomatic patients should also be educated about the signs and symptoms of TB and informed to present at a health facility immediately if these develop. - Asymptomatic child contacts of smear or culture positive MDR-TB should be managed according to the standard recommendations for drug susceptible TB. - Child contacts under 5-years of age should be given INH preventive therapy, irrespective of tuberculin response. - HIV-infected children of any age should be given INH preventive therapy, irrespective of tuberculin response. |
| Guidelines for the management of tuberculosis in children (6) | 2013 | - On the basis of the evidence that is currently available, WHO does not recommend second-line drugs for chemoprophylaxis in contacts of patients with MDR-TB. - However, for child contacts of patients with isoniazid mono-resistant TB, rifampicin 15mg/kg daily for 4 months may be given. Where the index patient has rifampicin mono-resistant TB, the child contact may be given isoniazid 10mg/kg daily for 6 months. |
| Management of drug-resistant tuberculosis – Policy guidelines (updated 2013) (7) | 2012 | - Managing Asymptomatic Contacts of MDR- and XDR-TB Patients: - The use of second-line drugs for preventive therapy in MDR- or XDR-TB contacts is not recommended. To date, no controlled clinical trials have been conducted to assess the efficacy of treatment for latent MDR- or XDR-TB infection. Close monitoring of asymptomatic patients for development of symptoms is therefore more appropriate, particularly in high TB burden settings where many different tubercle strains (most often drug-susceptible) are circulating. Given the real possibility that contacts may have been infected by drug-susceptible strains, it is acceptable practice to manage asymptomatic contacts of DR-TB patients in the same way as contacts of drug-susceptible TB patients. - Asymptomatic contacts of smear-negative MDR- and XDR-TB patients should be managed according to the standard recommendations for contacts of drug-susceptible TB patients. - Asymptomatic child contacts aged five years and younger and HIV-infected children irrespective of age should be considered for isoniazid preventive therapy. However, isoniazid preventive therapy often fails in these children. Therefore, regular two-monthly follow-up for symptoms (and CXR if indicated) should be done for first 6 months and 3-6 monthly thereafter for a minimum of two years. |

# Table B: Additional parameter inputs:

IGRA testing, TST, haematology and liver function tests are not routinely conducted to inform a decision to implement TPT in the <5 years age group (8). Testing for tuberculosis disease is recommended for child contacts in the 5 year and older age group prior to initiation of TPT (8), however, resource constraints mean that TST or IGRA is not routinely offered, therefore that are excluded from the baseline costs estimates. Therefore, these costs were not included in the base case analysis, but their inclusion enables calculation of costs under scenarios where additional tests are provided to subsets of the population at risk.

| TST | US$ 37.89 | (10) |
| --- | --- | --- |
| IGRA | US$ 109.08 | (11) |
| Haematology: full blood count | US$ 3.76 | (11) |
| Haematology: differential white cell count | US$ 2.06 | (11) |
| Liver function test: ALT | US$ 2.94 | (11) |
| Liver function test: BR | US$ 2.29 | (11) |

TST = Tuberculin Skin Testing IGRA = Interferon-Gamma Release Assay; ALT = Alanine aminotransferase; BL = Bilirubin

# Table C: Summary of patient pathway for TPT provision to household contacts:

The modelled pathway for representing costs for TPT initiation is representing in Figure 1 in the main manuscript. When MDR-TB is diagnosed, all household contacts exposed to the index case are identified and are requested to attend a screening visit at a primary health care clinic. The screening visit will include a clinical assessment, weight measurement, symptom screen, chest X-ray and HIV test where HIV status is unknown. While a tuberculin skin test (TST) is advised for HIV-negative children and an interferon gamma release assay (IGRA) is advised for HIV-positive children or for HIV-negative children if TST not available, a decision to administer TPT will be made independently of the diagnostic findings.

If there is no indication of TB disease, two management options are available:

(1) close clinical monitoring and continuous assessment of the household contact every three months in year one, then every six months in year two to monitor for development of TB disease, or
(2) administration of TPT followed by clinical monitoring up to two years after initial exposure.

If TPT is considered the optimal course of action, the child may undergo baseline haematology and liver function tests at the time of TPT initiation, but this is not a requirement for TPT. The appropriate TPT regimen will be selected based on the drug susceptibility test (DST) profile of the index case and can be initiated as soon as the resistance profile is known. Children taking TPT will attend monthly clinic visits for medicine collection and clinical monitoring (including clinical examination, routine blood tests and identification of adverse reactions). Children who discontinue or complete TPT attend the clinic every three months in year one, then every six months in year two after initial exposure to monitor for development of TB disease.

# Table D & Table E Weight banding in TB-CHAMP

The median weight-for-age z score for the n= 922 children and adolescents in the TB CHAMP trial was -0.4 (interquartile range -1.2 to 0.3) (12). The weight distribution observed in the TB-CHAMP trial in the 0-5 years age cohort is detailed in Table S4 below and was used to estimate average cost of weight-based dosing of TPT across the 0-5-years age group. The average age of each participant was calculated over the 6-month dosing period to accommodate for age variation as children gain weight over the course of their treatment.

Table D. Weight distribution for children aged <5 years (based on TB Champ participants <5 years of age)

| **Weight range (kg)** | **% in 0-1 year cohort** | **% in 2-4 year cohort** | **% in 0-5 year cohort** |
| --- | --- | --- | --- |
| 3 to 5.9 | 2.1% | 0.0% | 0.83% |
| 6 to 9.9 | 58.3% | 1.2% | 24.1% |
| 10 to 14.9 | 39.6% | 56.1% | 49.5% |
| 15 to 19.9 | 0.0% | 39.4% | 23.6% |
| 20 to 24.9 | 0.0% | 3.2% | 1.9% |
| 25 to 29.9 | 0.0% | 0.2% | 0.1% |
| 30 and over | 0.0% | 0.0% | 0.00% |

The TB CHAMP trial contained a relatively small number of children and adolescents in the 5-15 years age group (9%, n =83), which limited the ability to determine a representative weight distribution between the six weight band in the 5-15 years age group. Therefore, for this analysis, the ages of children in the 5 to 15-year range were aligned with weight categories based on WHO weight-for-age tables (13,14), applying similar methodology to Wilkinson et al. 2024 where WHO weight-for-age distributions were used to estimate costs of MDR/RR-TB treatment in adolescents and children (15).

Table E. Weight distribution for children aged 5-15 years (based on WHO weight-for-age tables)

| **Weight range (kg)** | **Corresponding months of life*** | **Proportion of population aged 5-15 years** |
| --- | --- | --- |
| 15 to 19.9 | m61-m69 | 7.5% |
| 20 to 24.9 | m70-m94 | 20.9% |
| 25 to 29.9 | m95-m114 | 16.7% |
| 30 to 34.9 | m115-m130 | 13.3% |
| 35 to 39.9 | m131-m143 | 10.8% |
| 40 to 44.9 | m144-m155 | 10.0% |
| 45 to 49.9 | m156-m167 | 10.0% |
| 50 and over | m168-m180 | 10.8% |
| All children aged 5-15 years | m61-m180 | 100% |

*Applying World Health Organization child growth standards (13,14)

# Table F: Health system costs (US$) by TPT regimen for children aged 0-<15 years (excluding pharmaceutical costs)

| **Management option** | **PHC screening costs** | **PHC TPT visit costs** | **PHC monitoring visit costs** | **Total** |
| --- | --- | --- | --- | --- |
| Clinical monitoring only^1^ | $82 | $0 | $83 | $165 |
| Six-month treatment options^2^  LFX; DLM; BDQ; 6H | $85 | $74 | $41 | $200 |
| Four month treatment option^3^  4R | $85 | $44 | $52 | $181 |
| Three month treatment options^4^  3HR; 3HP | $85 | $29 | $52 | $166 |

*BDQ-bedaquiline, DLM-delamanid, H- isoniazid, LFX-levofloxacin, P-rifapentine, R-rifampicin*

1. *After initial exposure: Close clinical monitoring every month for 6 months; then 3 monthly assessments up to one year; then 6 monthly assessments up to two years*
2. *After initial exposure: 6 months TPT; then 3 monthly assessments up to one year; then 6 monthly assessments up to two years. High dose isoniazid (6hH) indicated for RR/MDR-TB, standard dose isoniazid (stdH) indicated for drug-susceptible TB or rifampicin mono-resistant TB contact*
3. *After initial exposure: 4 months TPT; then 3 monthly assessments up to one year; then 6 monthly assessments up to two years*
4. *After initial exposure: 3 months TPT; then 3 monthly assessments up to one year; then 6 monthly assessments up to two years*
5. *Median household income based on reported household income of participants in the TB CHAMP*

Table G: Household costs (US$) (non-health system costs) by TPT regimen for children aged 0-<15 years

| **Management option** | **Direct household costs** | **Indirect household costs** | **Total household costs** | **Total as proportion of annual median household income** |
| --- | --- | --- | --- | --- |
| Clinical monitoring only | $12.29 | $27.19 | $39.49 | 1.8% |
| Six-month treatment options  LFX; DLM; BDQ; 6hH 6stdH | $14.80 | $32.73 | $47.53 | 2.1% |
| Four month treatment option  4R | $13.00 | $28.74 | $41.74 | 1.9% |
| Three month treatment options^4^  3HR; 3HP | $11.43 | $25.27 | $36.70 | 1.6% |

*BDQ-bedaquiline, DLM-delamanid, hH-high dose isoniazid, stdH-standard dose isoniazid, LFX-levofloxacin, P-rifapentine, R-rifampicin*

*Household cost estimations based on median costs as reported in MDRTBkids survey (16). Note for many people, out of pocket travel costs for PHC clinics are nil as survey data indicates it is common to walk, however there is often a significant time cost based on time to travel and wait at a PHC. Median annual household income (US$1,628) as reported by TB CHAMP households as part of collected household socioeconomic data at trial registration.*

# Table H: Pharmaceutical cost per patient (US$) completing TPT by regimen, formulation, weight band, and procurement mechanism

| **TPT regimen** | **Procurement mechanism*** | **3 to <6kg** | **6 to <10kg** | **10 to <15kg** | **15 to <20kg** | **20 to <25kg** | **25 to <30kg** | **30 to <35kg** | **35kg to <40kg** | **40 to <45kg** | **45 to <50kg** | **50kg and over** |
| --- | --- | --- | --- | --- | --- | --- | --- | --- | --- | --- | --- | --- |
| 6LFX_500mg | National | $11 | $11 | $11 | $22 | $22 | $22 | $22 | $22 | $22 | $22 | $33 |
|  | GDF | $4 | $4 | $4 | $7 | $7 | $7 | $7 | $7 | $7 | $7 | $11 |
| 6LFX_250mg | National | $8 | $16 | $16 | $24 | $24 | $32 | $32 | $32 | $32 | $32 | $48 |
|  | GDF | $2 | $4 | $4 | $7 | $7 | $9 | $9 | $9 | $9 | $9 | $13 |
| 6LFX_100mg | GDF | $10 | $29 | $38 | $48 | $57 | $67 | $95 | $95 | $95 | $95 | $95 |
| 6DLM_50mg | National | $278 | $278 | $278 | $278 | $278 | $278 | $556 | $835 | $1,113 | $1,113 | $1,113 |
|  | GDF | $243 | $243 | $243 | $243 | $243 | $243 | $486 | $730 | $973 | $973 | $973 |
| 6DLM_25mg | GDF | $582 | $582 | $582 | $582 | $582 | $582 | $1,164 | $1,745 | $2,327 | $2,327 | $2,327 |
| 6BDQ_100mg | National | $48 | $48 | $48 | $56 | $56 | $56 | $56 | $56 | $113 | $113 | $113 |
|  | GDF | $30 | $30 | $30 | $35 | $35 | $35 | $35 | $35 | $70 | $70 | $70 |
| 6BDQ_20mg | GDF | $22 | $45 | $78 | $117 | $117 | $196 | $196 | $196 | $391 | $391 | $391 |
| 6hH_100mg_tab | National | $2 | $6 | $8 | $12 | $12 | $17 | $17 | $19 | $19 | $25 | $25 |
|  | GDF | $1 | $3 | $3 | $5 | $5 | $7 | $7 | $8 | $8 | $10 | $10 |
| 6stdH_100mg_tab | National | $2 | $4 | $6 | $8 | $8 | $8 | $12 | $12 | $12 | $12 | $12 |
|  | GDF | $1 | $2 | $3 | $3 | $3 | $3 | $5 | $5 | $5 | $5 | $5 |
| 4R_150mg | National | $11 | $11 | $11 | $11 | $22 | $22 | $33 | $44 | $44 | $44 | $44 |
|  | GDF | $16 | $16 | $16 | $16 | $31 | $31 | $47 | $62 | $62 | $62 | $62 |
| 3HR (H 50mg / R 75mg and H 75mg/ R 150 mg) | National | $4 | $9 | $13 | $18 | $18 | $10 | $14 | $19 | $19 | $19 | $19 |
|  | GDF | $4 | $9 | $13 | $18 | $18 | $8 | $12 | $17 | $17 | $17 | $17 |
| 3HP (H 100mg / P 150 mg) | National | $4 | $6 | $8 | $11 | $15 | $15 | $19 | $23 | $23 | $23 | $24 |
|  | GDF | $4 | $5 | $7 | $11 | $15 | $15 | $18 | $22 | $22 | $22 | $22 |

BDQ-bedaquiline, DLM-delamanid, H-isoniazid, hH-high dose isoniazid, stdH-standard dose isoniazid, LFX-levofloxacin, P-rifapentine, R-rifampicin

*All TPT regimen formulations available under Global Drug Facility (GDF) procurement. Where a formulation is not listed under National Procurement, it is not available in South Africa under the national procurement mechanism (the Master Health Product List (MHPL))

# Table I: Total societal costs of different TPT regimen and formulation ($US)

| **Management option** | **Health system costs**  **(0-<15)** | **Pharmaceutical costs^1^** | | | | **Household costs**  **(0-<15)** | **Total societal costs** | | | |
| --- | --- | --- | --- | --- | --- | --- | --- | --- | --- | --- |
|  |  | **0-<2 years** | **2-<5 years** | **0-<5 years** | **5-<15 years** |  | **0-<2 years** | **2-<5 years** | **0-<5 years** | **5-<15 years** |
| Clinical monitoring only | $165 | $0 | $0 | $0 | $0 | $39 | $204 | $204 | $204 | $204 |
| 6LFX_500mg tab | $200 | $4 | $5 | $5 | $8 | $48 | $251 | $253 | $252 | $255 |
| 6LFX_250mg tab | $200 | $4 | $5 | $5 | $9 | $48 | $252 | $253 | $252 | $256 |
| 6LFX_100mg disp tab | $200 | $32 | $42 | $38 | $79 | $48 | $279 | $290 | $285 | $326 |
| 6LFX_optimised | $200 | $32 | $42 | $38 | $21 | $48 | $279 | $290 | $285 | $269 |
| 6DLM_50mg tab | $200 | $243 | $243 | $243 | $553 | $48 | $490 | $490 | $490 | $800 |
| 6DLM_25mg disp tab | $200 | $582 | $582 | $582 | $1,322 | $48 | $829 | $829 | $829 | $1,569 |
| 6DLM_optimised | $200 | $582 | $581 | $581 | $649 | $48 | $829 | $828 | $829 | $896 |
| 6BDQ_100mg tab | $200 | $30 | $32 | $31 | $46 | $48 | $277 | $279 | $278 | $293 |
| 6BDQ_20mg tab | $200 | $58 | $95 | $80 | $233 | $48 | $305 | $342 | $327 | $481 |
| 6BDQ_optimised | $200 | $58 | $94 | $80 | $69 | $48 | $305 | $342 | $327 | $316 |
| 6hH_100mg tab | $200 | $3 | $4 | $4 | $7 | $48 | $250 | $251 | $251 | $254 |
| 6stdH_100mg tab | $200 | $2 | $3 | $3 | $4 | $48 | $249 | $250 | $250 | $252 |
| 4R_150mg cap | $181 | $16 | $16 | $16 | $45 | $42 | $238 | $238 | $238 | $267 |
| 3HR_FDC tab | $166 | $11 | $15 | $13 | $15 | $37 | $213 | $218 | $216 | $218 |
| 3HP_100mg + 150mg tab | $166 | $6 | $9 | $8 | $18 | $37 | $209 | $211 | $210 | $220 |

*BDQ-bedaquiline, DLM-delamanid, hH-high dose isoniazid, stdH-standard dose isoniazid, LFX-levofloxacin, P-rifapentine, R-rifampicin*

1. *Global Drug Facility procurement prices used for pharmaceutical costs. Health system non-pharmaceutical costs and Household costs are not impacted by pharmaceutical procurement mechanism.*

# Figure A: Average pharmaceutical costs across weight bands for children <5 years for TPT regimen and formulation by procurement mechanism

*BDQ-bedaquiline, DLM-delamanid, hH-high dose isoniazid, stdH-standard dose isoniazid, LFX-levofloxacin, P-rifapentine, R-rifampicin.*

*Tab = tablet formulation.*

U*nder optimised regimen: children weighing <25kg children receive dispersible formulation, children weighing 25kg and over receive solid dosage formulation. LFX 100mg dispersible tablet, DLM 25mg dispersible tablet and BDQ 20 mg dispersible tablet not available under South African national procurement at time of publication*

# Figure B: Pharmaceutical cost (US$) of TPT regimen and formulation by weight for children <5 years of age using Global Drug Facility procurement pricing

*BDQ-bedaquiline, H-isoniazid, hH-high dose isoniazid, stdH-standard dose isoniazid, LFX-levofloxacin, P-rifapentine, R-rifampicin. Average is mean cost by weight distribution in the 0- <5 year age group.*

References

1.	National Department of Health Republic of South Africa. Standard Treatment Guidelines and Essential Medicines List for South Africa: Hospital level paediatrics. 2023 Edition. Pretoria: Department of Health; 2023. Available from: http://www.health.gov.za/

2.	Department of Health. National guidelines on the treatment of tuberculosis infection. Pretoria; 2023.

3.	South African Department of Health. Management of rifampicin-resistant tuberculosis: A clinical reference guide. Pretoria; 2019.

4.	National Department of Health Republic of South Africa. Standard Treatment Guidelines and Essential Medicines List for South Africa: Hospital level paediatrics. 2017 Edition. Pretoria: Department of Health; 2017.

5.	Department of Health. National Tuberculosis Management Guidelines 2014 . Pretoria; 2014. Available from: https://www.knowledgehub.org.za/elibrary/national-tuberculosis-management-guidelines

6.	National Department of Health. Guidelines for the management of tuberculosis in children 2013. Pretoria; 2013.

7.	Department of Health. Management of drug-resistant tuberculosis - Policy guidelines (updated 2013). Pretoria; 2012.

8.	World Health Organization. WHO operational handbook on tuberculosis. Module 1: Prevention. Tuberculosis preventive treatment. Second edition. Geneva; 2024.

9.	World Health Organization. WHO operational handbook on tuberculosis. Module 5: management of tuberculosis in children and adolescents. Geneva; 2022.

10.	Mandalakas AM, Hesseling AC, Gie RP, Schaaf HS, Marais BJ, Sinanovic E. Modelling the cost-effectiveness of strategies to prevent tuberculosis in child contacts in a high-burden setting. Thorax. 2013;68(3):247–55.

11.	National Health Laboratory Service. NHLS State price list 2023/24. Sandringham; 2023.

12.	Hesseling AC, Purchase SE, Martinson NA, Fairlie L, Schaaf HS, Brigden J, et al. Levofloxacin Preventive Treatment in Children Exposed to MDR Tuberculosis. New England Journal of Medicine. 2024 Dec 19;391(24):2315–26.

13.	World Health Organization. WHO growth reference data (children aged 5-19 years) . Geneva: World Health Organization; 2007. Available from: https://www.who.int/tools/growth-reference-data-for-5to19-years

14.	World Health Organization. WHO child growth standards (children aged 0-5 years) . Geneva: World Health Organization; 2006. Available from: https://www.who.int/tools/child-growth-standards

15.	Wilkinson T, Garcia-Prats AJ, Sachs T, Paradkar M, Suryavanshi N, Kinikar A, et al. Pharmaceutical cost dynamics for the treatment of rifampicin-resistant tuberculosis in children and adolescents in South Africa, India, and the Philippines. PLoS One. 2024 Jul 1;19.

16.

16.	Wilkinson T, Hesseling AC, Hoddinott G, Anthony MG, Schaaf HS, Sinanovic E, et al. Household costs and health-related quality of life of childhood multidrug-resistant TB in Western Cape, South Africa. IJTLD Open. 2026 Jul 13;3(7):435–43.
