# Supplementary material for: Health system and household costs of preventive treatment delivery for children exposed to multidrug-resistant tuberculosis in South Africa
Source: PLoS One. 2026 Oct 8;21(10):e0336561. doi: 10.1371/journal.pone.0336561 (PMC13648985; doi:10.1371/journal.pone.0336561)
Supplement: Supplementary file 2 [file pone.0336561.s002.docx]

This study is providing important information on household and health system related cost of TB preventive treatment in South African context. Study findings provide important insights on key cost drivers that can influence national policy making. Data included up to date pharmaceutical prices. Household cost and system cost perspective provide comprehensive understanding of economic burden of TPT in South Africa.

I have few minor comments.

Introduction:

Line 12-16: flow looks abrupt from “lack of robust data” to evidence has now emerged…. It is important if author can elaborate what kind of evidence.

Line 35: TB treatment at the point of “ use” replace it with “care” as in previously it was written as point of care.

Line 73: Adult formulation of the Levofloxacin formulation. Formulation word is repeated twice.

Methods:

Line 75 to 77: TB-CHAMP evaluated Levofloxacin VS Placebo and no other TPT options so extrapolating its adverse event profile to all options is not clear to me and may need further justification. Additionally, study included children below 5 years of age but model applies same adverse event assumption across all age groups.

Lines 129-131: How an adverse drug reaction cost are estimated. Have you taken into consideration multiple clinic visits due to ADRs?

When estimating system level cost have you considered drug storage cost, supply chain cost, stock management cost and loss of drug due to expiry.

Line 152-153: When estimating household cost whether author considered in analysis cost of visiting a facility in urban Vs Rural area, by type of facility, Primary health center Vs tertiary care center.

Line 186-188: There may also be possibility of generic entry of Bedaquiline and Delamanid which may reduce the cost of these drugs and hence the statement “*Potential future TPT regimens of delamanid and bedaquiline, if proven to be efficacious and safe,*

*187 have substantially higher cost than levofloxacin”* need to re-drafted

Discussion:

Though study align with prior Global and multicounty studies it lacks generalizability beyond South Africa. Empirical inputs are based on clinical trial settings and may differ from program implementation. Author have included all these aspects in discussions and limitation.
