# Supplementary material for: Health system and household costs of preventive treatment delivery for children exposed to multidrug-resistant tuberculosis in South Africa
Source: PLoS One. 2026 Oct 8;21(10):e0336561. doi: 10.1371/journal.pone.0336561 (PMC13648985; doi:10.1371/journal.pone.0336561)
Supplement: Supplementary file 3 [file pone.0336561.s003.docx]

**Reviewer comments and author responses****:**

**“Health system and household costs of preventive treatment delivery for children exposed to multidrug-resistant tuberculosis in South Africa” (PONE_D_25_58170)**

**Editorial**

| **No** | **Comment** | **Response** |
| --- | --- | --- |
| E.1 | The manuscript presents a valuable economic model regarding the costs of delivering TPT for children. However, the current draft lacks sufficient clarity regarding its methodology and overall flow. | Thank you, further specific responses below. |
| E.2 | The manuscript and abstract currently create confusion regarding the distinction between this study and the TB-CHAMP trial. While the introduction discusses this to some extent, the authors must explicitly discuss this in both the Abstract and Methods sections (or avoid mentioning TB-CHAMP trial in abstract). | Thank you for drawing this to our attention and we agree the original phrasing of the abstract was confusing. We have revised the abstract and reviewed the methods section of the manuscript to ensure this is clear. |
| E.3 | Add objective of the study | We have revised text to state the objective in the methods section |
| E.4 | The Methods section requires structing. Start with clearly define the study design, the type of study, and its specific how its related to the TB-CHAMP trial.  Add study context and setting thereafter- As health systems and costs vary drastically across different settings and countries, the authors must describe the specific health system and setting where this study was conducted. Considering global audience, please include details on the TB program and healthcare infrastructure, health insurance and patient costs, and human resources.  I highly suggest utilizing the established patient pathway (Figure 1) more effectively within the main text to provide a breakdown of the specific costs involved at each stage of TPT implementation. | Thank you for this constructive suggestion. We agree that the Methods section could be more clearly structured for an international readership. We have therefore reorganised the Methods to more explicitly distinguish the study design, analytical approach, relationship to the TB-CHAMP trial, and the South African health system context. We have also added clearer sub-sections describing pharmaceutical, health system, and household costing approaches, and strengthened integration of the patient pathway (Figure 1) within the main text to clarify the resource utilisation and cost components represented at each stage of TPT delivery. |
| E.5 | Please detail the sources of data collection and the specific types of costs analyzed. Add comments on whether the cost data was validated through data triangulation or if it was based on expert consensus. | Thank you for this comment. We agree that clear description of data sources and costing approach is important. All cost inputs used in the analysis are derived from empirically observed data or published price sources and are fully referenced in the main text and supplementary materials (Table 1 and associated appendices).  Specifically, pharmaceutical prices were obtained from national procurement data and the Global Drug Facility catalogue, health service unit costs from published literature and national fee schedules, and laboratory costs from the National Health Laboratory Service price list. Household costs were derived from primary data collected in the MDRTBkids survey. Resource utilisation parameters (e.g. clinic visits, adherence) were informed by empirical data from the TB-CHAMP trial and national guidelines.  The analysis did not rely on expert elicitation or consensus-based estimates for cost parameters. Where assumptions were required (e.g. patient pathway and visit frequency), these were based on national guidelines and clearly stated.  To improve clarity for readers, we have strengthened the description of data sources and costing approach in the Methods section and ensured that all key inputs are clearly signposted to their corresponding references. |
| E.6 | As implementing TPT services requires a massive upscaling effort for already overburdened TB healthcare providers, yet the current model excludes these service delivery costs. The authors should estimate the cost of programmatic implementation—specifically contact tracing and the household visits required to identify eligible children—to reflect the true financial burden of scaling this program. | We agree that implementation of a national-scale TPT programme would involve additional programme-level and health system costs beyond the per-patient service delivery costs included in this analysis. However, the objective of the present study was to estimate the per-patient costs associated with different TPT regimens and formulations, rather than to undertake a full programmatic budget impact analysis of national TPT scale-up.  A national programme budget impact analysis would require a substantially broader costing framework, including estimation of costs associated with contact tracing systems, community and household outreach, programme management and supervision, pharmacy and logistics systems, medicine storage and distribution, health workforce expansion, training, infrastructure utilisation, and monitoring and evaluation systems. Such costs are also likely to vary substantially between settings depending on existing TB programme structure and implementation model.  We have clarified this distinction further in the Methods and Discussion. We consider that the primary contribution of the present analysis is to provide transparent and transferable per-patient cost estimates to inform comparison, prioritisation, and future economic evaluation of different TPT regimen options for children exposed to MDR-TB. |
| E.7 | Greater clarity and justification are required regarding the assumptions made about adverse events. Assuming a "zero cost" for regimens involving bedaquiline and delamanid is not valid and should be addressed. | We have clarified the Methods section to make it clear that it is not the intent to extrapolate an assumption of no adverse events to other potential TPT regimen. We show the direct cost differences associated with different potential regimen as a comparison to levofloxacin (see also earlier response to a similar comment). Text has been included to clarify this point and note that future analysis of other potential TPT candidates should incorporate adverse event rates when these become empirically available in addition to all the other required inputs to accurately cost other regimen, including specific weight-based dosing, length of treatment, and adherence information. |
| E.8 | Additionally, I suggest adding the primary trial reference (Hesseling et al.) after every mention of the TB-CHAMP trial throughout the manuscript to maintain consistent citation standards. | Thank you for this suggestion. We agree that the linkage between the costing analysis and the primary TB-CHAMP trial publication should be clear. We have ensured that the primary TB-CHAMP publication (Hesseling et al.) is cited at key first mentions and in sections where empirical trial-derived parameters are discussed throughout the manuscript. |
| E.9 | I also suggest to limit the discussion on TPT adherence as it might not be the objective of this study. | Thank you for this suggestion. We agree that the primary focus of the manuscript is costing rather than adherence outcomes. References to adherence have therefore been limited to areas where they directly influence interpretation of the costing analysis. Specifically, adherence is discussed to contextualise the use of empirically observed medicine utilisation from the TB-CHAMP trial and to acknowledge that adherence under trial conditions may differ from routine implementation. We also briefly discuss adherence in relation to formulation choice, as the TB-CHAMP trial primarily utilised the 250 mg tablet formulation and adherence and acceptability may differ if child-friendly dispersible formulations are more widely available in programmatic settings. We have revised the Discussion to ensure this framing is clearer and more concise |

**Reviewer #1:**

| **No.** | **Comment** | **Author response** |
| --- | --- | --- |
| **1.1** | **Overall:**  Thank you for the opportunity to review this manuscript on costs incurred by the health system and households during TPT delivery for children exposed to MDR-TB in South Africa. This is a well thought and executed analysis, and the manuscript is clearly written. An important addition to the existing literature. | Thank you, specific responses below. |
| 1.2 | Methods:  “*..may be appropriate for estimating and implementation budget impact analysis” -* probably not clear or a typo in this | Thank you, agreed. Corrected in the text. |
| 1.3 | “Healthcare costs associated with TPT provision excluded procedures and interventions required for the identification and management of TB disease and/or those required for the management and monitoring of HIV”. For children who develop symptoms or disease during TPT or clinical monitoring only, doesn't this exclusion result in gross underestimation of the true costs of a decision: giving TPT or not? | Thank you for this important comment. The costs of diagnosing and managing TB disease were considered outside the scope of this analysis, which was designed as a direct costing study of TPT and monitoring provision rather than an evaluation of the full clinical pathway following MDR/RR-TB exposure. We agree that children who develop symptoms or TB disease during monitoring or TPT may incur additional healthcare costs. However, incorporating these downstream costs would also require inclusion of the costs of TB disease occurring outside routine monitoring, together with the corresponding health outcomes associated with different management approaches. We consider that these questions are more appropriately addressed within a cost-effectiveness framework rather than a direct costing analysis, noting also that the total rate of TB disease observed in the trial was low (n=17 cases of TB disease by week 48 out of n = 922 total participants) (1). We have clarified this distinction and limitation in the Discussion. |
| 1.4 | “The analysis estimated household costs by applying the average per-instance cost of attending clinic visits and collecting prescribed medicines as observed in the MDRTBkids survey..”. While I agree this is a reasonable approximation, I wonder if the time spent attending clinics would be the same given the scope of activities done during those visits? Would a sensitivity analysis help with this? | We agree that the duration and activities undertaken during clinic visits for TPT may differ from those observed in the MDRTBkids survey, which was conducted among households of children receiving MDR-TB treatment. We therefore acknowledge that the per-visit household costs applied in this analysis represent an approximation. However, household costs were estimated using a simple multiplication of per-visit costs and expected numbers of visits, meaning that any variation in visit duration or associated opportunity costs would have a linear and readily predictable effect on the overall estimates. Given the relatively small contribution of household costs to total societal costs and the absence of empirical data describing household costs specifically associated with TPT visits, we did not undertake a formal sensitivity analysis. We have clarified this limitation in the Discussion. |
| 1.5 | I presume most of the parameters used are uncertain - was this accounted for? Would be useful to present results and their corresponding uncertainties. | We agree that uncertainty should be considered when interpreting costing analyses. However, the majority of inputs in this study were based on actual observed prices, tariffs, or guideline-specified dosing schedules rather than statistically estimated parameters. For example, pharmaceutical costs were derived from published procurement prices, laboratory costs from national price lists, and dosing schedules from the most recent WHO and national guidance. Consequently, the primary sources of uncertainty in this analysis relate to structural assumptions, such as procurement mechanism, formulation choice, regimen selection, and weight-based dosing, rather than parameter uncertainty around estimated values. We sought to address these sources of uncertainty by presenting costs across multiple procurement scenarios, formulations, regimens, and paediatric weight bands. We have clarified this distinction in the Discussion. |
| 1.6 | *Results*  Please provide a textual summary of the “Total societal costs of TPT provision” in Figure 3. | Thank you for this suggestion. We agree that the Results section should explicitly summarise the key findings presented in Figure 3. We have added a textual summary of the total societal costs by regimen, including the range of total costs observed and the principal drivers of variation between regimens. |
| 1.7 | “Pharmaceutical costs represent the largest variation…”. Please state the ranges. | Thank you, we agree. The pharmaceutical cost range has now been added to the Results section. |
| 1.8 | “with non-pharmaceutical health system costs relatively stable between options (US$165 for clinical monitoring only to US$200 for 6-month regimen with monitoring)”. Not entirely sure I understand this. Perhaps rephrase? It’s also difficult to determine the absolute cost contributions on the figure, so I suggest adding a table even in the appendix as I think these are important. | Thank you – a note has been added after this statement directing readers to Table 8 in the Appendix |
| 1.9 | “As a sensitivity analysis, the total health system costs for the <5 age groups increase to $307 and $236 depending on whether an IGRA or TST is used…”. Which regimen does this refer to or is this some weighted average? Are these results shown in a Table/Figure? | We agree that the wording was unclear. The analysis was intended as a descriptive scenario analysis illustrating the impact of adding either an IGRA or TST to the screening pathway. Because the cost of these tests is additive and independent of TPT regimen choice, the increase in health system costs would apply similarly across all regimens. We have revised the text to clarify this and avoid the impression that these estimates relate to a specific regimen or weighted average. |
| 1.10 | *Discussion*  “Costs specific to the operational aspects of the TB CHAMP were excluded..but it is possible that some aspects of the clinical trial influenced outcomes such as adherence. This should be taken into consideration in any secondary analysis utilising costs parameters in this analysis.”. This is an important point and I do not see why this was not investigated in a sensitivity analysis since the authors had access to these data. | We agree that clinical trial conditions may influence participant behaviour, and service utilisation. However, our concern was not primarily that trial-specific operational activities altered the unit costs included in the analysis, but rather that the trial environment may have influenced factors such as adherence, treatment completion, and attendance at scheduled visits (2) (3). These effects may arise from aspects of trial participation that are difficult to quantify, including enhanced follow-up, participant engagement, and increased support from dedicated research personnel. While some trial-specific costs were excluded from analysis, we do not consider it possible to robustly parameterise the behavioural effects of trial participation in a sensitivity analysis using the available data. We acknowledge that the original wording in the manuscript did not clearly convey this distinction and have revised the Discussion to clarify this as a limitation and that the concern relates to the influence of the trial environment on participant behaviour rather than uncertainty in the underlying cost estimates. |
| 1.11 | The authors correctly stated that “Contact tracing to identify children for TPT initiation can have substantial human resource costs including community health workers, administrators and clinicians and should be incorporated if assessing a wider prevention programme.”, but it’s not clear why this was stated. I think they want to mention this was not included in the analysis - and they should do so. | We agree that the original wording did not state clearly enough that contact management and case-finding costs were excluded from the present analysis. The intention of this statement was to distinguish between the per-patient costs of TPT provision estimated in this study and the wider costs of operationally implementing a prevention programme at population scale. We have revised the Discussion to explicitly state that contact management, household outreach, and case-finding activities were not included in the analysis and would be important considerations in a broader programme costing or budget impact analysis. recommendation about doing this in future research? |
| 1.12 | “...involves crushing the tablet and suspending in water, yoghurt, or other palatable media…”. This is an important point that warrants further investigation but I acknowledge this maybe outside the scope of the current study - but might be worth emphasising that. Perhaps could be included as part of patient cost surveys. | Thank you for this helpful suggestion. We agree that administration of different formulations may have implications for caregiver time, effort, and treatment acceptability that were not captured in the present analysis. While this was outside the scope of the current study, we have expanded the Discussion to highlight this as an important area for future household cost and implementation research. In parallel, we have analysed the acceptability of the formulation and manipulation used in the trial (250 mg levofloxacin and matched placebo) using both quantitative and qualitative methods. |
| 1.13 | The exclusion of adverse events associated costs on the basis of results from the TB-CHAMP trial - which is fair - however it may not be sufficient to simply say “Secondary analysis or use of the costs should acknowledge this assumption and add costs of adverse event management if adverse events of TPT are found to be significant in future.” Why was this not investigated in this modelling study which clearly presents the opportunity to do so? And is the exclusion valid for all regimens given TB-CHAMP only evaluated levofloxacin? In the methods, the authors state that “Monthly TPT dispensing visits consisted of a standard clinic visit, checking for adverse drug reactions, …”. With the exclusion of these costs, I wonder if it’s worth dropping the visits altogether or reducing the scope of things done during the visit as a way of reducing costs? | Thank you for this important comment. We agree that adverse event–related costs are a relevant component of cost analyses. In the TB-CHAMP trial, no meaningful differences in adverse events were observed between levofloxacin and placebo arms and overall rates of adverse events were low (grade 3 or higher adverse event rate n = 4/453 in levofloxacin arm and n=8/497 in placebo arm, HR 0.52, 95% CI 0.16 – 1.71) (1). We have also added text to the manuscript noting subsequent pooled analysis of the TB-CHAMP and V-QUIN trials found that serious and grade 3 or higher adverse events were uncommon and did not differ significantly between levofloxacin and placebo overall, although adverse events resulting in treatment discontinuation and musculoskeletal adverse events were more frequently reported in older age groups, particularly adolescents and adults.. (4).  In the absence of robust comparative empirical data for alternative regimens, incorporating adverse event costs directly into the base-case analysis would require multiple speculative assumptions. These would include assumptions about the probability and severity distribution of adverse events, and with different regimens associated resource use (e.g. primary care visits, hospitalisation, diagnostics, and treatment), and the potential impact on adherence and treatment completion. Such layered assumptions risk introducing additional uncertainty and bias into the estimates.  We agree that this assumption is most directly supported for levofloxacin, the regimen evaluated in TB-CHAMP, and is more uncertain for alternative regimens included for comparison. For this reason, we have strengthened the discussion of this limitation and clarified that future analyses should incorporate regimen-specific adverse event costs where empirical data become available. The Phoenix trial comparing delamanid vs. isoniazid will provide efficacy, safety and health economics data on alternative MDR-TPT regimens (5). Results are expected in 2027.  With respect to monitoring visits, these were retained because they reflect current clinical practice and guideline-based monitoring requirements rather than solely management of adverse events. The routine clinic visit was assumed to include adverse event screening as one component of a broader clinical assessment including dose adjustment by weight band in growing young children. Consequently, exclusion of adverse event treatment costs does not imply that the monitoring visit itself should be removed from the pathway.  In response to this and similar reviewer comments, we have also added a descriptive scenario analysis illustrating how overall costs may change under plausible assumptions regarding adverse event–related resource utilisation, without introducing unsupported parameterisation into the model. |

**Reviewer #2:**

| **No.** | **Comment** | **Author response** |
| --- | --- | --- |
| 2.1 | Overall: This manuscript provides important insights to health system and household level costs of TB preventive treatment delivery to children exposed to MDR-TB. It is written in technically sound manner with rigorous statistical analysis. It followed ethical principles and authors reported having all data made available in Public domain. | Thank you. |

**Reviewer #3:**

| **No.** | **Comment** | **Response** |
| --- | --- | --- |
| 3.0 | *Overall:* This study aims to assesses the cost of delivering TPT for children <15 years exposed to MDR-TB from a health system and societal perspective in the South African context, reflecting differences in costs associated with weight-based dosing, formulations, and procurement mechanisms. This is a good study provide the information for TB program for preparing the implementation of TPT for children < 15 years who are MDR/RR contact. However, the authors may need to clarify some issues as mentioned below: | Thank you, specific responses below. |
| 3.1 | Please clarify MDR-TB or MDR/RR TB in the manuscript. For example, in practice, children are contacted to MDR or Rifampicin resistance (RR) TB, not only MDR-TB. In the lines 21-22, “The findings from TB-CHAMP and V-QUIN have informed the 2024 World Health Organization (WHO) recommendation for a six-month levofloxacin regimen for household contacts of people with MDR-TB (11). And in the ref #11clearly mentioned “… in contacts exposed to multidrug- or rifampicin-resistant tuberculosis” | We agree that current WHO recommendations relate to contacts exposed to MDR/RR-TB rather than MDR-TB alone. For simplicity, the manuscript primarily uses the term MDR-TB; however, we recognise that this may be interpreted inconsistently. We have therefore added text in the introduction section, clarifying that, throughout the manuscript, MDR-TB is used broadly to refer to MDR/RR-TB exposure, consistent with current preventive treatment recommendations and programmatic management. |
| 3.2 | In my opinion, in order to estimate the cost of delivering TPT for children <15 years exposed to MDR/RR-TB, the authors should based on the algorithm of screening and delivery TPT which are currently doing (for example, with drug-sensitivity household contacted) by NTP, or follow WHO’s recommendation. In the practice, it’s very difficult to follow the study condition (TB-CHAMP). So that, please clarify in the figure 1, care pathway of MDR-TB contacts, have process of initiate close clinical monitoring, which requested PHC clinic monitoring visit every 3 months for 2 years from initial exposure. Do this process have implemented for any TPT delivery and monitoring in TB program or have been recommended by WHO? If not, please consider to excluded or at least separate the cost analysis for with or without PHC clinic monitoring visit every 3 months for 2 years from initial exposure. | We agree that routine programmatic implementation of TPT may involve less intensive follow-up than was assumed in the TB-CHAMP protocol given the fact that is was a randomized double blinded placebo-controlled trial and that regular clinical monitoring for two years following exposure may not occur in all settings. The objective of this analysis was to estimate the costs associated with a clearly defined care pathway, with all cost components transparently reported so that National TB Programmes can adapt the estimates to local implementation arrangements.  To address this concern, we have clarified in the manuscript that monitoring costs were based on four scheduled primary healthcare clinic visits and may not reflect all programmatic settings. We have also added an illustrative scenario analysis demonstrating the impact of reduced monitoring intensity on overall costs. This showed that reducing monitoring from four to two clinic visits would reduce total health system costs by approximately 12%, while the relative differences between TPT regimens remain largely unchanged. We therefore retained the original costing assumptions but have made the underlying resource use and its impact on costs more explicit to facilitate adaptation to local policies and practice. |
| 3.3 | Table 1, please check the cost of Chest X-ray (US$ 66.64), ref # 32 | Thank you, yes this has been checked and we confirm this is the estimation of the health system cost of a chest x-ray in US$, and the source reference is correct as this has been referenced from a prior economic evaluation that directly identified the cost of an x-ray in South African hospitals.. |
| 3.4 | Figure 3, please check why the household cost is very high in DLM regimens. Which items in household cost made this cost is very high in DLM regimens. | Household costs do not differ substantially between regimens. The apparent difference in Figure 3 reflects the scale of the figure and the substantially higher health-system costs associated with DLM itself, which is a major cost driver, rather than a meaningful difference in household costs. We have clarified this in the Results. |

**Reviewer #4:**

| **No.** | **Comment** | **Response** |
| --- | --- | --- |
| 4.1 | This is an important addition to the literature - the cost of TPT, if prohibitive, could undermine the gains made in the field of TPT as countries face competing healthcare priorities - it is thus important to evaluate the most cost-effective interventions to help tackle the TB burden in children and reduce the fatality due to MDR-TB in children. | Thank you, specific responses below. |
| 4.2 | If TB treatment in SA is free, what costs (direct and indirect ) make up the $500 household costs stated in the lit review in the Background section, and does this current analysis reveal that? The results sections suggest that the current analysis show much lower HH costs. Can authors comment on this discrepancy? | The approximately $500 household costs reported in the background section reflected costs associated with treatment of disease. Our analysis presented here reflects costs associated with preventive treatment (i.e one drug over 6 months) only. Text is added in discussion to reflect the lower costs to households associated with prevention compared to treatment. |
| 4.3 | Costs incorporated the adverse event rate observed in the CHAMP trial. Did the authors consider any variability (range of values) around this or rates observed in other studies? This is an important evaluation to make. | Thank you for this important comment. We agree that adverse event–related costs are a relevant component of cost analyses. In the TB-CHAMP trial, no meaningful differences in adverse events were observed between arms and overall rates adverse events rates were very low. In the absence of robust comparative empirical data for alternative regimens, incorporating adverse event costs directly into the base-case analysis would require multiple speculative assumptions. These would include assumptions about the probability and severity distribution of adverse events, associated resource use (e.g. primary care visits, hospitalisation, diagnostics, and treatment), and the potential impact on adherence and treatment completion. Such layered assumptions risk introducing additional uncertainty and bias into the estimates.  We therefore did not include adverse event cost and management in the base-case analysis. However, in response to this comment, we have added a descriptive scenario analysis in the Discussion to illustrate the potential impact of adverse events on overall costs under plausible assumptions (e.g. additional primary care visits or short hospitalisations). This approach allows us to demonstrate the magnitude of potential cost changes without introducing unsupported parameterisation into the model.  We have clarified this rationale in the Methods and expanded the Discussion to highlight this limitation and outline how future analyses could incorporate adverse event–related resource use where robust empirical data become available. Please also refer to out other responses to similar comments by other reviewers. |
| 4.4 | The rate of clinic visits –these two sentences seem contradictory. The rate of scheduled medicine dispensing and monitoring visits attended by participants in the TB-CHAMP trial were used to reflect cost implications expected during implementation. AND Expected frequency of routine clinic visits were calculated based on South Africa’s national TB guidance (20). | Thank you, agree. We have adjusted the text to clarify that the rate of adherence to scheduled visits was inferred from the TB CHAMP trial, while the number of modelled clinic visits were informed by national TB guidance. |
| 4.5 | Can the authors explain the reasons why LF 250 mg costs are higher than 500mg under the national procurement pricing? This does not seem to be the case in the GDF pricing. The discussion mentions that this formulation is cheaper under GDF but does not explain the difference (locally) between 500 and 250 mg. Are there differences in, say, clinical monitoring, adverse events requiring further clinical interventions, etc.? | The difference in cost between the 250mg and 500mg are attributable to the differing unit costs, and potential for halving tablets to achieve the required weight-based dosing, as detailed in the appendix. Relative differences between the 250mg and 500mg formulations under national procurement (tender process) and GDF pricing is due to relative unit prices only, this has been clarified in the manuscript text. |
| 4.6 | Can the authors also discuss under what policy/political conditions/decisions could enable SA to procure through GDF | Thank you for this suggestion. We agree that the policy context surrounding procurement is important when interpreting the GDF pricing scenarios. We have expanded the Discussion to note that access to GDF pricing would depend on national procurement policies and decisions regarding the use of international pooled procurement mechanisms, and that future procurement reforms or purchasing arrangements could influence the feasibility of obtaining these prices as well as sustained source of WHO prequalified formulations without stockouts and formulation switches. |

**Reviewer # (Word document attached to decision email from PLOS One)**

| **No.** | **Comment** | **Response** |
| --- | --- | --- |
| #.1 | This study is providing important information on household and health system related cost of TB preventive treatment in South African context. Study findings provide important insights on key cost drivers that can influence national policy making. Data included up to date pharmaceutical prices. Household cost and system cost perspective provide comprehensive understanding of economic burden of TPT in South Africa | Thank you, further specific responses below. |
| #.2 | *Introduction:* Line 12-16: flow looks abrupt from “lack of robust data” to evidence has now emerged…. It is important if author can elaborate what kind of evidence. | Thank you agreed, sentence has been edited to make it clear that the evidence referred to is the TB CHAMP and VQUIN trials. |
| #.3 | Line 35: TB treatment at the point of “ use” replace it with “care” as in previously it was written as point of care. | Thank you, change made. |
| #.4 | Line 73: Adult formulation of the Levofloxacin formulation. Formulation word is repeated twice. | Thank you, change made. |
| #.5 | *Methods:* Line 75 to 77: TB-CHAMP evaluated Levofloxacin VS Placebo and no other TPT options so extrapolating its adverse event profile to all options is not clear to me and may need further justification. Additionally, study included children below 5 years of age, but model applies same adverse event assumption across all age groups. | Thank you for this important comment. We agree that the adverse event profile observed in TB-CHAMP, which enrolled children aged <5 years, may not be directly generalisable to older children and adolescents. However, adverse event costs were not explicitly modelled in the base-case analysis for any age group or regimen. We have clarified this limitation in the Discussion and note that future analyses incorporating adverse event costs should consider age-specific and regimen-specific evidence where available. |
| #.6 | Lines 129-131: How an adverse drug reaction cost are estimated. Have you taken into consideration multiple clinic visits due to ADRs? | Thank you for this comment. As the TB CHAMP trial did not identify a statistically significant adverse event profile, costs of adverse events were not included in the base case. We have described in the text how the cost profile would change assuming increased risk of adverse events requiring additional clinic visits and hospital stays. |
| #.7 | When estimating system level cost have you considered drug storage cost, supply chain cost, stock management cost and loss of drug due to expiry. | Thank you for this important comment. We agree that wider health system costs such as medicine storage, supply chain management, stock management systems, and losses associated with expiry may be relevant when estimating the costs of implementing a national-scale TPT programme. However, the present analysis focused on estimating direct per-patient service delivery costs associated with different TPT regimens and formulations, rather than broader programme-level implementation or system overhead costs. We have clarified this distinction further in the Methods and Discussion. We agree that inclusion of these additional system costs would be appropriate in future programme budgeting or implementation analyses. |
| #.8 | Line 152-153: When estimating household cost whether author considered in analysis cost of visiting a facility in urban Vs Rural area, by type of facility, Primary health center Vs tertiary care center. | Household cost estimates in this analysis were informed by the MDRTBkids household survey, which predominantly reflected urban and peri-urban households accessing primary healthcare services in the Western Cape, South Africa. We agree that household costs associated with accessing TPT may differ in rural settings, where travel distances, transport costs, and time requirements may be greater. We have clarified this in the Methods and added this as a limitation in the Discussion. |
| #.9 | Line 186-188: There may also be possibility of generic entry of Bedaquiline and Delamanid which may reduce the cost of these drugs and hence the statement “*Potential future TPT regimens of delamanid and bedaquiline, if proven to be efficacious and safe, have substantially higher cost than levofloxacin”* need to re-drafted | We agree and have redrafted the text as suggested. |
| #.10 | *Discussion:* Though study align with prior Global and multicounty studies it lacks generalizability beyond South Africa. Empirical inputs are based on clinical trial settings and may differ from program implementation. Author have included all these aspects in discussions and limitation. | We agree that transferability of the results beyond South Africa requires consideration of differences in health system structure, procurement mechanisms, and implementation context. We also acknowledge that empirical utilisation inputs derived from the TB-CHAMP trial may differ from routine programmatic implementation. These considerations are now more explicitly highlighted in the Discussion and limitations section. |

**References**

1.	Hesseling AC, Purchase SE, Martinson NA, Fairlie L, Schaaf HS, Brigden J, et al. Levofloxacin Preventive Treatment in Children Exposed to MDR Tuberculosis. New England Journal of Medicine. 2024 Dec 19;391(24):2315–26. doi:10.1056/NEJMOA2314318 PubMed PMID: 39693542.

2.	Purchase SE, Brigden J, Seddon JA, Martinson NA, Fairlie L, Staples S, et al. Acceptability of a 250 mg levofloxacin formulation in children receiving TB preventive treatment. IJTLD open. 2025;2(3):129–36.

3.	Purchase SE, Wademan DT, Tshetu NL, Rafique M, Hoddinott G, Seddon JA, et al. Holistic acceptability of an adult levofloxacin formulation in children and adolescents on a tuberculosis preventive treatment trial. PLOS Global Public Health. 2024;4(7):e0003381.

4.	Duong T, Brigden J, Simon Schaaf H, Garden F, Marais BJ, Anh Nguyen T, et al. A Meta-Analysis of Levofloxacin for Contacts of Multidrug-Resistant Tuberculosis. NEJM Evidence. 2025 Jan;4(1). doi:10.1056/EVIDOA2400190 PubMed PMID: 39693627.

5.	Kendall MA, Hughes MD, Kim S, Aaron LA, Naini L, Suryavanshi N, et al. Protecting households on exposure to newly diagnosed index multidrug-resistant tuberculosis patients: Study protocol for the PHOENIx phase 3 clinical trial. Contemp Clin Trials. 2025 Nov 1;158:108084. doi:10.1016/J.CCT.2025.108084 PubMed PMID: 40992540.
