# Supplementary material for: Health system and household costs of preventive treatment delivery for children exposed to multidrug-resistant tuberculosis in South Africa
Source: PLoS One. 2026 Oct 8;21(10):e0336561. doi: 10.1371/journal.pone.0336561 (PMC13648985; doi:10.1371/journal.pone.0336561)
Supplement: Peer review history

# Peer review: Decision Letter 0

**DOI:** 10.1371/journal.pone.0336561.r001

**Authors:** Yasir Alvi

24 Mar 2026

Dear Dr. Wilkinson,

Thank you for submitting your manuscript to PLOS ONE. After careful consideration, we feel that it has merit but does not fully meet PLOS ONE’s publication criteria as it currently stands. Therefore, we invite you to submit a revised version of the manuscript that addresses the points raised during the review process.

==============================

The manuscript presents a valuable economic model regarding the costs of delivering TPT for children. However, the current draft lacks sufficient clarity regarding its methodology and overall flow.

The manuscript and abstract currently create confusion regarding the distinction between this study and the TB-CHAMP trial. While the introduction discusses this to some extent, the authors must explicitly discuss this in both the Abstract and Methods sections (or avoid mentioning TB-CHAMP trial in abstract).

Add objective of the study

The Methods section requires structing. Start with clearly define the study design, the type of study, and its specific how its related to the TB-CHAMP trial.

Add study context and setting thereafter- As health systems and costs vary drastically across different settings and countries, the authors must describe the specific health system and setting where this study was conducted. Considering global audience, please include details on the, TB program and healthcare infrastructure, health insurance and patient costs, and human resources.

Please detail the sources of data collection and the specific types of costs analyzed. Add comments on whether the cost data was validated through data triangulation or if it was based on expert consensus.

I highly suggest utilizing the established patient pathway (Figure 1) more effectively within the main text to provide a breakdown of the specific costs involved at each stage of TPT implementation.

As implementing TPT services requires a massive upscaling effort for already overburdened TB healthcare providers, yet the current model excludes these service delivery costs. The authors should estimate the cost of programmatic implementation—specifically contact tracing and the household visits required to identify eligible children—to reflect the true financial burden of scaling this program.

Greater clarity and justification are required regarding the assumptions made about adverse events. Assuming a "zero cost" for regimens involving bedaquiline and delamanid is not valid and should be addressed.

Additionally, I suggest adding the primary trial reference (Hesseling et al.) after every mention of the TB-CHAMP trial throughout the manuscript to maintain consistent citation standards.

I also suggest to limit the discussion on TPT adherence as it might not be the objective of this study.

==============================

Please submit your revised manuscript by May 08 2026 11:59PM. If you will need more time than this to complete your revisions, please reply to this message or contact the journal office at plosone@plos.org. When you're ready to submit your revision, log on to https://www.editorialmanager.com/pone/ and select the 'Submissions Needing Revision' folder to locate your manuscript file.

- A letter that responds to each point raised by the academic editor and reviewer(s). You should upload this letter as a separate file labeled 'Response to Reviewers'.
- A marked-up copy of your manuscript that highlights changes made to the original version. You should upload this as a separate file labeled 'Revised Manuscript with Track Changes'.
- An unmarked version of your revised paper without tracked changes. You should upload this as a separate file labeled 'Manuscript'.

If you would like to make changes to your financial disclosure, please include your updated statement in your cover letter. Guidelines for resubmitting your figure files are available below the reviewer comments at the end of this letter.

If applicable, we recommend that you deposit your laboratory protocols in protocols.io to enhance the reproducibility of your results. Protocols.io assigns your protocol its own identifier (DOI) so that it can be cited independently in the future. For instructions see: https://journals.plos.org/plosone/s/submission-guidelines#loc-laboratory-protocols. Additionally, PLOS ONE offers an option for publishing peer-reviewed Lab Protocol articles, which describe protocols hosted on protocols.io. Read more information on sharing protocols at https://plos.org/protocols?utm_medium=editorial-email&utm_source=authorletters&utm_campaign=protocols.

As the corresponding author, your ORCID iD is verified in the submission system and will appear in the published article. PLOS supports the use of ORCID, and we encourage all coauthors to register for an ORCID iD and use it as well. Please encourage your coauthors to verify their ORCID iD within the submission system before final acceptance, as unverified ORCID iDs will not appear in the published article. *Only*  the individual author can complete the verification step; PLOS staff *cannot*  verify ORCID iDs on behalf of authors.

We look forward to receiving your revised manuscript.

Kind regards,

Yasir Alvi

Academic Editor

PLOS One

Journal Requirements:

When submitting your revision, we need you to address these additional requirements.

1.Please ensure that your manuscript meets PLOS ONE's style requirements, including those for file naming. The PLOS ONE style templates can be found at

https://journals.plos.org/plosone/s/file?id=wjVg/PLOSOne_formatting_sample_main_body.pdf and

https://journals.plos.org/plosone/s/file?id=ba62/PLOSOne_formatting_sample_title_authors_affiliations.pdf

2. Thank you for stating the following financial disclosure:

“Supported by Unitaid through a grant (2019-36-SUN-MDR to Stellenbosch University) from the BENEFIT Kids project; the Joint Global Health Trials program through a grant (MR/M007340/1 to Stellenbosch University) funded by several U.K. agencies (the Department of Health and Social Care; the Foreign, Commonwealth, and Development Office; the Global Challenges Research Fund; the Medical Research Council [MRC]; and Wellcome) as part of the European and Developing Countries Clinical Trials Partnership 2 program supported by the European Union; a grant (to Stellenbosch University) from the South African Medical Research Council; a grant (to Dr. Hesseling) from the South African National Research Foundation; and grants (MC_UU_00004/04 and MC_UU_00004/09 to the MRC Clinical Trials Unit at University College London) from the MRC”

Please state what role the funders took in the study.  If the funders had no role, please state: "The funders had no role in study design, data collection and analysis, decision to publish, or preparation of the manuscript."

If this statement is not correct you must amend it as needed.

Please include this amended Role of Funder statement in your cover letter; we will change the online submission form on your behalf.

3. Please provide a complete Data Availability Statement in the submission form, ensuring you include all necessary access information or a reason for why you are unable to make your data freely accessible. If your research concerns only data provided within your submission, please write "All data are in the manuscript and/or supporting information files" as your Data Availability Statement.

4. Please include captions for your Supporting Information files at the end of your manuscript, and update any in-text citations to match accordingly. Please see our Supporting Information guidelines for more information: http://journals.plos.org/plosone/s/supporting-information.

5. If the reviewer comments include a recommendation to cite specific previously published works, please review and evaluate these publications to determine whether they are relevant and should be cited. There is no requirement to cite these works unless the editor has indicated otherwise.

[Note: HTML markup is below. Please do not edit.]

Reviewer's Responses to Questions

**Comments to the Author**

1. Is the manuscript technically sound, and do the data support the conclusions?

Reviewer #1: Yes

Reviewer #2: Yes

Reviewer #3: Yes

Reviewer #4: Yes

**********

2. Has the statistical analysis been performed appropriately and rigorously?

Reviewer #1: No

Reviewer #2: Yes

Reviewer #3: N/A

Reviewer #4: Yes

**********

3. Have the authors made all data underlying the findings in their manuscript fully available?

The PLOS Data policy

Reviewer #1: Yes

Reviewer #2: Yes

Reviewer #3: Yes

Reviewer #4: Yes

**********

4. Is the manuscript presented in an intelligible fashion and written in standard English?

Reviewer #1: Yes

Reviewer #2: Yes

Reviewer #3: Yes

Reviewer #4: Yes

**********

Reviewer #1: Health system and household costs of preventive treatment delivery for children exposed to multidrug-resistant tuberculosis in South Africa

Overview

Thank you for the opportunity to review this manuscript on costs incurred by the health system and households during TPT delivery for children exposed to MDR-TB in South Africa. This is a well thought and executed analysis, and the manuscript is clearly written. An important addition to the existing literature. A few comments/suggestions for consideration.

Comments

Methods

“..may be appropriate for estimating and implementation budget impact analysis” - probably not clear or a typo in this

“Healthcare costs associated with TPT provision excluded procedures and interventions required for the identification and management of TB disease and/or those required for the management and monitoring of HIV.”. For children who develop symptoms or disease during TPT or clinical monitoring only, doesn't this exclusion result in gross underestimation of the true costs of a decision: giving TPT or not?

“The analysis estimated household costs by applying the average per-instance cost of attending clinic visits and collecting prescribed medicines as observed in the MDRTBkids survey..”. While I agree this is a reasonable approximation, I wonder if the time spent attending clinics would be the same given the scope of activities done during those visits? Would a sensitivity analysis help with this?

I presume most of the parameters used are uncertain - was this accounted for? Would be useful to present results and their corresponding uncertainties.

Results

Please provide a textual summary of the “Total societal costs of TPT provision” in Figure 3.

“Pharmaceutical costs represent the largest variation…”. Please state the ranges.

“with non-pharmaceutical health system costs relatively stable between options (US$165 for clinical monitoring only to US$200 for 6-month regimen with monitoring)”. Not entirely sure I understand this. Perhaps rephrase? It’s also difficult to determine the absolute cost contributions on the figure, so I suggest adding a table even in the appendix as I think these are important.

“As a sensitivity analysis, the 206 total health system costs for the <5 age groups increase to $307 and $236 depending on whether an IGRA or TST is used…”. Which regimen does this refer to or is this some weighted average? Are these results shown in a Table/Figure?

Discussion

“Costs specific to the operational aspects of the TB CHAMP were excluded..but it is possible that some aspects of the clinical trial influenced outcomes such as adherence. This should be taken 309 into consideration in any secondary analysis utilising costs parameters in this analysis.”. This is an important point and I do not see why this was not investigated in a sensitivity analysis since the authors had access to these data.

The authors correctly stated that “Contact tracing to identify children for TPT initiation can have substantial human resource costs including community health workers, administrators and clinicians and should be incorporated if assessing a wider prevention programme.”, but it’s not clear why this was stated. I think they want to mention this was not included in the analysis - and they should do so.

“...involves crushing the tablet and suspending in water, yoghurt, or other palatable media…”. This is an important point that warrants further investigation but I acknowledge this maybe outside the scope of the current study - but might be worth emphasising that. Perhaps could be included as part of patient cost surveys.

The exclusion of adverse events associated costs on the basis of results from the TB-CHAMP trial - which is fair - however it may not be sufficient to simply say “Secondary analysis or use of the costs should acknowledge this assumption and add costs of adverse event management if adverse events of TPT are found to be significant in future.”. Why was this not investigated in this modelling study which clearly presents the opportunity to do so? And is the exclusion valid for all regimens given TB-CHAMP only evaluated levofloxacin? In the methods, the authors state that “Monthly TPT dispensing visits consisted of a standard clinic visit, checking for adverse drug reactions, …”. With the exclusion of these costs, I wonder if it’s worth dropping the visits altogether or reducing the scope of things done during the visit as a way of reducing costs?

Reviewer #2: This manuscript provide important insights to health system and household level costs of TB preventive treatment delivery to children exposed to MDR-TB. It is written in technically sound manner with rigorous statistical analysis. It followed ethical principles and authors reported having all data made available in Public domain.

Reviewer #3: This study aims to assesses the cost of delivering TPT for children <15 years exposed to MDR-TB from a health system and societal perspective in the South African context, reflecting differences in costs associated with weight-based dosing, formulations, and procurement mechanisms. This is a good study provide the information for TB program for preparing the implementation of TPT for children < 15 years who are MDR/RR contact. However, the authors may need to clarify some issues as mentioned below:

1. Please clarify MDR-TB or MDR/RR TB in the manuscript. For example, in practice, children are contacted to MDR or Rifampicin resistance (RR) TB, not only MDR-TB. In the lines 21-22, “The findings from TB-CHAMP and V-QUIN have informed the 2024 World Health Organization (WHO) recommendation for a six-month levofloxacin regimen for household contacts of people with MDR-TB (11). And in the ref #11clearly mentioned “… in contacts exposed to multidrug- or rifampicin-resistant tuberculosis”

2. In my opinion, in order to estimate the cost of delivering TPT for children <15 years exposed to MDR/RR-TB, the authors should based on the algorithm of screening and delivery TPT which are currently doing (for example, with drug-sensitivity household contacted) by NTP, or follow WHO’s recommendation. In the practice, it’s very difficult to follow the study condition (TB-CHAMP). So that, please clarify in the figure 1, care pathway of MDR-TB contacts, have process of initiate close clinical monitoring, which requested PHC clinic monitoring visit every 3 months for 2 years from initial exposure. Do this process have implemented for any TPT delivery and monitoring in TB program or have been recommended by WHO? If not, please consider to excluded or at least separate the cost analysis for with or without PHC clinic monitoring visit every 3 months for 2 years from initial exposure.

3. Table 1, please check the cost of Chest X-ray (US$ 66.64), ref # 32

4. Figure 3, please check why the household cost is very high in DLM regimens. Which items in household cost made this cost is very high in DLM regimens.

Reviewer #4: Summary

This study evaluated costs of levofloxacin (recently WHO-recommended for children exposed to MDR TB); comparing the costs to a) potential future TPT options for MDR-TB exposure and b)DS-TB TPT options.

Cost data were collected as part of the TB-CHAMP a phase III a trial to evaluate six months of daily levofloxacin versus placebo in children in South Africa. The cost evaluation was done from the health system and societal/household perspectives

This is an important addition to the literature - the cost of TPT, if prohibitive, could undermine the gains made in the field of TPT as countries face competing healthcare priorities - it is thus important to evaluate the most cost-effective interventions to help tackle the TB burden in children and reduce the fatality due to MDR-TB in children.

The authors used the patient-care pathway and assigned costs (health systems and household) along this in order to derive total costs.

N=45 households surveyed.

In my review I considered: how well the study was executed in terms of the data sources, statistical and general analytical methods, the clarity of presentation and the discussion points that demonstrate how this study can help shape policy.

Specific comments

• If TB treatment in SA is free,what costs (direct and indirect ) make up the $500 household costs stated in the lit review in the Background section, and does this current analysis reveal that? The results sections suggest that the current analysis show much lower HH costs. Can authors comment on this discrepancy?

• Costs incorporated the adverse event rate observed in the CHAMP trial. Did the authors consider any variability (range of values) around this or rates observed in other studies? This is an important evaluation to make.

• The rate of clinic visits –these two sentences seem contradictory

o The rate of scheduled medicine dispensing and monitoring visits attended by participants in the TB-CHAMP trial were used to reflect cost implications expected during implementation. AND Expected frequency of routine clinic visits were calculated based on South Africa’s national TB guidance (20).

• Can the authors explain the reasons why LF 250 mg costs are higher than 500mg under the national procurement pricing? This does not seem to be the case in the GDF pricing. The discussion mentions that this formulation is cheaper under GDF but does not explain the difference (locally) between 500 and 250 mg. Are there differences in, say, clinical monitoring, adverse events requiring further clinical interventions, etc.?

• Can the authors also discuss under what policy/political conditions/decisions could enable SA to procure through GDF

**********

what does this mean?). If published, this will include your full peer review and any attached files.

If you choose “no”, your identity will remain anonymous but your review may still be made public.

**Do you want your identity to be public for this peer review?**  For information about this choice, including consent withdrawal, please see our Privacy Policy

Reviewer #1: **Yes:**  Nyashadzaishe Mafirakureva

Reviewer #2: No

Reviewer #3: No

Reviewer #4: No

**********

[NOTE: If reviewer comments were submitted as an attachment file, they will be attached to this email and accessible via the submission site. Please log into your account, locate the manuscript record, and check for the action link "View Attachments". If this link does not appear, there are no attachment files.]

To ensure your figures meet our technical requirements, please review our figure guidelines: https://journals.plos.org/plosone/s/figures

You may also use PLOS’s free figure tool, NAAS, to help you prepare publication quality figures: https://journals.plos.org/plosone/s/figures#loc-tools-for-figure-preparation.

NAAS will assess whether your figures meet our technical requirements by comparing each figure against our figure specifications.

Attachment Submitted filename: Reviewers comments.docx

# Author comment: Author response to Decision Letter 1

**DOI:** 10.1371/journal.pone.0336561.r002

14 Jun 2026

Thank you for the detailed reviewer comments and editorial comments. We trust these have all been addressed and we refer to the Response to Reviewer uploaded document.

Attachment Submitted filename: Responses to reviewers PONE_D_25_58170 TPT costing final.docx

# Peer review: Decision Letter 1

**DOI:** 10.1371/journal.pone.0336561.r003

**Authors:** Yasir Alvi

1 Sep 2026

Health system and household costs of preventive treatment delivery for children exposed to multidrug-resistant tuberculosis in South Africa

PONE-D-25-58170R1

Dear Dr. Wilkinson,

We’re pleased to inform you that your manuscript has been judged scientifically suitable for publication and will be formally accepted for publication once it meets all outstanding technical requirements.

Within one week, you’ll receive an e-mail detailing the required amendments. When these have been addressed, you’ll receive a formal acceptance letter and your manuscript will be scheduled for publication.

An invoice will be generated when your article is formally accepted. Please note, if your institution has a publishing partnership with PLOS and your article meets the relevant criteria, all or part of your publication costs will be covered. Please make sure your user information is up-to-date by logging into Editorial Manager at Editorial Manager® and clicking the ‘Update My Information' link at the top of the page. For questions related to billing, please contact billing support.

If your institution or institutions have a press office, please notify them about your upcoming paper to help maximize its impact. If they’ll be preparing press materials, please inform our press team as soon as possible -- no later than 48 hours after receiving the formal acceptance. Your manuscript will remain under strict press embargo until 2 pm Eastern Time on the date of publication. For more information, please contact onepress@plos.org.

Kind regards,

Yasir Alvi

Academic Editor

PLOS One

Reviewers' comments:

Reviewer's Responses to Questions

**Comments to the Author**

Reviewer #2: All comments have been addressed

Reviewer #4: All comments have been addressed

**********

2. Is the manuscript technically sound, and do the data support the conclusions?

Reviewer #2: Yes

Reviewer #4: Yes

**********

3. Has the statistical analysis been performed appropriately and rigorously?

Reviewer #2: Yes

Reviewer #4: Yes

**********

4. Have the authors made all data underlying the findings in their manuscript fully available?

The PLOS Data policy

Reviewer #2: Yes

Reviewer #4: Yes

**********

5. Is the manuscript presented in an intelligible fashion and written in standard English?

Reviewer #2: Yes

Reviewer #4: Yes

**********

Reviewer #2: Recommended for publication, as the reviewers' comments have been addressed appropriately by the authors.

Reviewer #4: All comments have been addressed. Thank you for addressing all the comments. this is an important addition to the literature on the economic value of TPT for children.

**********

what does this mean?). If published, this will include your full peer review and any attached files.

If you choose “no”, your identity will remain anonymous but your review may still be made public.

**Do you want your identity to be public for this peer review?**  For information about this choice, including consent withdrawal, please see our Privacy Policy

Reviewer #2: **Yes:**  Nishi Suryavanshi

Reviewer #4: No

**********

# Editor report: Acceptance letter

**DOI:** 10.1371/journal.pone.0336561.r004

**Authors:** Yasir Alvi

PONE-D-25-58170R1

PLOS One

Dear Dr. Wilkinson,

I'm pleased to inform you that your manuscript has been deemed suitable for publication in PLOS One. Congratulations! Your manuscript is now being handed over to our production team.

At this stage, our production department will prepare your paper for publication. This includes ensuring the following:

* All references, tables, and figures are properly cited

* All relevant supporting information is included in the manuscript submission,

* There are no issues that prevent the paper from being properly typeset

You will receive further instructions from the production team, including instructions on how to review your proof when it is ready. Please keep in mind that we are working through a large volume of accepted articles, so please give us a few days to review your paper and let you know the next and final steps.

Lastly, if your institution or institutions have a press office, please let them know about your upcoming paper now to help maximize its impact. If they'll be preparing press materials, please inform our press team within the next 48 hours. Your manuscript will remain under strict press embargo until 2 pm Eastern Time on the date of publication. For more information, please contact onepress@plos.org.

You will receive an invoice from PLOS for your publication fee after your manuscript has reached the completed accept phase. If you receive an email requesting payment before acceptance or for any other service, this may be a phishing scheme. Learn how to identify phishing emails and protect your accounts at https://explore.plos.org/phishing.

If we can help with anything else, please email us at customercare@plos.org.

Thank you for submitting your work to PLOS One and supporting open access.

Kind regards,

PLOS One Editorial Office Staff

on behalf of

Dr. Yasir Alvi

Academic Editor

PLOS One
